# Preserved structural connectivity mediates the clinical effect of thrombolysis in patients with anterior-circulation stroke

**DOI:** 10.1038/s41467-021-22786-w

**Published:** 2021-05-10

**Authors:** Eckhard Schlemm, Thies Ingwersen, Alina Königsberg, Florent Boutitie, Martin Ebinger, Matthias Endres, Jochen B. Fiebach, Jens Fiehler, Ivana Galinovic, Robin Lemmens, Keith W. Muir, Norbert Nighoghossian, Salvador Pedraza, Josep Puig, Claus Z. Simonsen, Vincent Thijs, Anke Wouters, Christian Gerloff, Götz Thomalla, Bastian Cheng

**Affiliations:** 1 Klinik und Poliklinik für Neurologie, Kopf- und Neurozentrum, University Medical Center Hamburg-Eppendorf, Hamburg, Germany; 2 Hospices Civils de Lyon, Service de Biostatistique, Lyon, France; 3 Université Lyon 1, Villeurbanne, France; 4 CNRS, UMR 5558, Laboratoire de Biométrie et Biologie Evolutive, Equipe Biostatistique-Santé, Villeurbanne, France; 5 Centrum für Schlaganfallforschung Berlin (CSB), Charité - Universitätsmedizin Berlin, Campus Mitte, Berlin, Germany; 6 Klinik für Neurologie, Medical Park Berlin Humboldtmühle, Berlin, Germany; 7 Klinik und Hochschulambulanz für Neurologie, Charité-Universitätsmedizin Berlin, Campus Mitte, Berlin, Germany; 8 German Center for Neurodegenerative Diseases (DZNE), Berlin, Germany; 9 German Centre for Cardiovascular Research (DZHK), Berlin, Germany; 10 ExcellenceCluster NeuroCure, Berlin, Germany; 11 Department of Diagnostic and Interventional Neuroradiology, University Medical Center Hamburg-Eppendorf, Hamburg, Germany; 12 Department of Neurology, University Hospitals Leuven, Leuven, Belgium; 13 KU Leuven – University of Leuven, Department of Neurosciences, Experimental Neurology, Leuven, Belgium; 14 VIB, Center for Brain & Disease Research, Laboratory of Neurobiology, Campus Gasthuisberg, Leuven, Belgium; 15 Institute of Neuroscience & Psychology, University of Glasgow, Glasgow, UK; 16 Department of Stroke Medicine, Université Claude Bernard Lyon 1, CREATIS CNRS UMR 5220-INSERM U1206, INSA-Lyon; Hospices Civils de Lyon, Lyon, France; 17 Department of Radiology, Institut de Diagnostic per la Image (IDI), Hospital Dr Josep Trueta, Institut d’Investigació Biomèdica de Girona (IDIBGI), Parc Hospitalari Martí i Julià de Salt - Edifici M2, Salt, Girona, Spain; 18 Department of Neurology, Aarhus University Hospital, Aarhus, Denmark; 19 Stroke Division, Florey Institute of Neuroscience and Mental Health, University of Melbourne, Heidelberg, VIC Australia; 20 Austin Health, Department of Neurology, Heidelberg, VIC Australia

**Keywords:** Network models, Stroke

## Abstract

Thrombolysis with recombinant tissue plasminogen activator in acute ischemic stroke aims to restore compromised blood flow and prevent further neuronal damage. Despite the proven clinical efficacy of this treatment, little is known about the short-term effects of systemic thrombolysis on structural brain connectivity. In this secondary analysis of the WAKE-UP trial, we used MRI-derived measures of infarct size and estimated structural network disruption to establish that thrombolysis is associated not only with less infarct growth, but also with reduced loss of large-scale connectivity between grey-matter areas after stroke. In a causal mediation analysis, infarct growth mediated a non-significant 8.3% (CI_95%_ [−8.0, 32.6]%) of the clinical effect of thrombolysis on functional outcome. The proportion mediated jointly through infarct growth and change of structural connectivity, especially in the border zone around the infarct core, however, was as high as 33.4% (CI_95%_ [8.8, 77.4]%). Preservation of structural connectivity is thus an important determinant of treatment success and favourable functional outcome in addition to lesion volume. It might, in the future, serve as an imaging endpoint in clinical trials or as a target for therapeutic interventions.

## Abstract (web summary)

The effects of thrombolysis therapy in stroke on structural connectivity are not well understood. Here, the authors show with causal mediation analysis that the clinical benefits of thrombolysis in anterior circulation stroke are mediated via structural connectivity.

## Introduction

Systemic thrombolysis with recombinant tissue plasminogen activator is an effective treatment for acute ischemic stroke^1,2^. Its beneficial effect on clinical outcome is commonly attributed to the attenuation of growth of irreversible tissue damage after recanalization of an arterial occlusion. However, it remains an open question which imaging biomarker of preserved brain structure is most suited for explaining the favorable effects of reperfusion and for making accurate predictions of clinical outcome.

One common surrogate marker for tissue loss is the volume of a stroke lesion as measured by restricted diffusion on magnetic resonance imaging (MRI), or hypodensity or impaired perfusion on computed tomography (CT). In the absence of reperfusion, stroke lesions grow in the first hours and days after stroke onset^3–5^ due to the conversion of areas with reversible ischemia to infarction. Secondary analyses of large clinical trials indicate that alteplase reduces lesion growth in short- and long-term follow-ups ranging from 24 h to 3 months^6–8^. In a pooled analysis, attenuation of lesion growth by thrombolytic therapy was associated with favorable clinical outcome^9–11^. However, there are exceptions to this general trend. In the recent DEFUSE-3 trial, the clinical benefit of arterial recanalization was not accompanied by large statistically significant differences of absolute lesion volumes or growth between the treatment group and control group on follow-up imaging 24 h after stroke^12^. A similar observation was made in the WAKE-UP trial (Efficacy and Safety of MRI-Based Thrombolysis in WAKE-UP Stroke). In WAKE-UP, patients with unknown time of stroke onset were randomized to alteplase or placebo based on an MRI selection criterion^13^. Treatment with alteplase was effective in terms of a favorable clinical outcome measured by a modified Rankin scale (mRS) score of 0–1 points. While significantly higher rates of favorable outcome were observed in the alteplase group, this was not associated with smaller stroke lesion volumes 22–36 h after stroke compared to placebo.

These findings indicate that factors other than the size of stroke lesions may be relevant for the therapeutic effect of thrombolytic therapy. In particular, lesion location determines neurological deficits, and spatial patterns of lesion distribution are linked to clinical outcome^14^. Furthermore, beyond focal damage, stroke lesions impair neurological functions by disrupting the large-scale spatiotemporal organization of neuronal activity in the brain^15^. The physical substrate for this is the structural connectome, representing anatomical connections in the brain. Stroke lesions frequently occur in the cerebral white matter, disrupting essential pathways of the structural connectome that support and shape normal brain function^16^. These disruptions can be quantified using graph-theoretical measures of large-scale network topology^17–19^. Complementarily, structural disconnection can be characterized by loss-of-connectivity profiles of pre-specified cortical and subcortical grey-matter regions. The pattern and extent of structural disconnections depend on the size and anatomical localization of causative stroke lesions and determine the nature of functional deficits^20^ and clinical recovery after stroke^21^ beyond measures of focal damage. The effects of systemic thrombolysis on structural connectivity changes after stroke have not yet been examined.

We aimed to investigate changes in the structural connectome after thrombolysis using data from a large prospective, randomized, multicentre trial of thrombolysis in acute ischemic stroke. In this secondary analysis of the WAKE-UP trial (ClinicalTrials.gov number NCT01525290, EudraCT number 2011-005906-32), stroke lesions were projected onto reference tractograms from healthy subjects to determine their effect on the structural connectivity profile. We hypothesized that treatment with alteplase would attenuate the loss of structural connectivity and that preserved connectivity, in addition to reduced growth of the ischemic lesion, would contribute to mediating the treatment effect of systemic thrombolysis.

## Results

### Patient characteristics

The composition of the final study population resulting from the application of inclusion and exclusion criteria is shown in Supplementary Fig. 1. Of 503 patients randomized in the WAKE-UP trial to receive placebo or alteplase, 352 had MR imaging data of acceptable quality both before randomization and 22–36 h after stroke. Of those, 278 (79.0%) had an anterior-circulation stroke (territory supplied by the anterior or middle cerebral artery; 160 lesions in the left hemisphere, 109 lesions in the right hemisphere, 9 bilateral); 61 (17.3%) had a posterior-circulation stroke (25 PCA, 7 cerebellum, 29 brain stem) and 13 (3.7%) had signs of acute cerebral ischemia in more than one vascular territory.

Of the 269 patients thus included in the study, 142 were randomized to placebo and 127 to alteplase. Age (placebo: mean 66.0 ± 11.1 years; alteplase: mean 65.2 ± 11.3 years) was comparable between the two groups, while baseline NIHSS scores (placebo: median 7, interquartile range [4, 11]; alteplase: median 6, IQR [3.5, 8]) showed a tendency toward higher values in the placebo group.

Median duration from pre-randomization MRI to follow-up imaging was 26:03 h (IQR [24:22, 29:03] hours). Spatial distribution of stroke lesions is shown in Supplementary Fig. 2.

Of the 151 patients excluded from analysis because of insufficient image quality, 119 had anterior-circulation infarcts. Their age (66.4 ± 11.7 years) and baseline NIHSS scores (median 6, IQR [4, 10.5]) were similar to the study population.

### Treatment effects and clinical outcome

In 6 of 269 patients (2.2%, 2 placebo, 4 alteplase), the primary endpoint was missing. Of the remaining patients, 131 (49.8%) reached the primary endpoint of modified Rankin scale score 0 or 1. This favorable outcome was achieved in 58 patients in the placebo group (41.4%) and in 73 patients in the alteplase group (59.3%). In a logistic regression analysis, alteplase was associated with a higher probability of favorable clinical outcome (odds ratio [OR] adjusted for age, baseline NIHSS score and pre-randomization lesion volume 1.98, 95%-confidence interval [CI_95%_] [1.13, 3.51], *P* = 0.018).

### Stroke lesion volumes

In the study population, median lesion volume on diffusion-weighted imaging (DWI) before randomization was 3.0 ml (IQR [1.0, 9.9] ml). After 22–36 h, median lesion volumes, measured on FLAIR imaging, had increased to 4.4 ml ([1.4, 22] ml), which was significant in a Wilcoxon signed-rank test (*P* < 2 × 10^−16^). In the placebo group, lesion volume increased from 4.0 ml (IQR [1.3, 10] ml) to 5.5 ml (IQR [1.7, 25] ml). In the thrombolysis group, infarcts grew from 2.2 ml (IQR [0.87, 9.9] ml) to 3.4 ml (IQR [1.1, 20] ml) as illustrated in Supplementary Fig. 3. Absolute lesion volumes did not differ between placebo and alteplase groups before randomization (*P* = 0.139), but at follow-up were smaller in the thrombolysis group (*P* = 0.044). Larger increases of infarct size were observed in the placebo group (median 2.3 ml; IQR [0.24, 17] ml) compared to patients treated with alteplase (median 1.0 ml; [−0.27, 7.5] ml). The difference was statistically significant (Mann–Whitney U test, two-sided *P* = 0.018). This interaction was confirmed in a linear-mixed effects model of the temporal evolution of lesion volume as well as in a simple linear regression of lesion volume at T2 against treatment allocation with lesion volume at T1 included as a nuisance regressor (Supplementary Table 1). Adjusted for age, baseline NIHSS score, baseline lesion volume and treatment allocation, higher infarct growth was strongly associated with a lower probability of achieving a favorable functional outcome: the odds ratio associated with a doubling of the growth ratio vol^T2^/vol^T1^ was 0.77 (CI_95%_ [0.62, 0.93], *P* 0.009).

### Systemic thrombolysis modifies the progression of network disruption after stroke

**Fig. 1:**
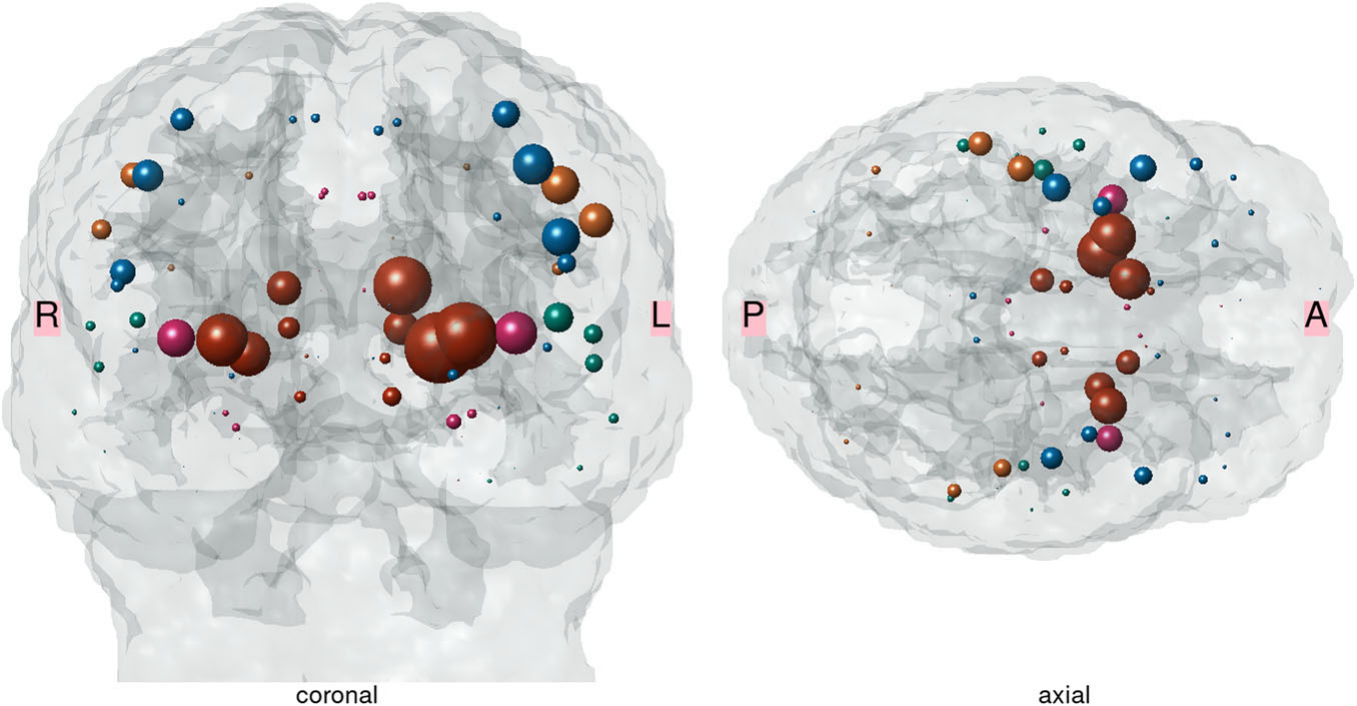
Pattern and magnitude of structural disconnection induced by acute ischemic stroke lesions prior to randomization illustrated on a three-dimensional template in standard MNI space. Lesions were segmented on diffusion-weighted images (DWI) and mapped onto reference tractograms from 73 healthy subjects using the NeMo Toolbox. Spheres are located at the center of gravity of each of 86 brain regions of the Desikan–Killiany parcellation. Volume indicates magnitude of average change of connectivity with larger sizes indicating higher degree of disconnection. Colors indicate assignment of brain regions to frontal (cerulean), parietal (sun), temporal (dark cyan), occipital (summer sky) and limbic (violet red) lobes, or subcortical structures (orange). In the coronal section, the brain template is shown using the neurological convention. This Figure was created using the NeMo toolbox^39^.

Pattern and magnitude of network disruptions induced by the initial ischemic lesions, quantified by the Change of Connectivity (ChaCo) score, are illustrated in Fig. 1. In this score, values of zero indicate that reconstructed fiber bundles originating from a grey-matter area do not pass through the stroke region at all (no disconnection), while the maximal value of one represents complete disconnection of a piece of cortex or subcortical nucleus from the rest of the network.

The propensity of brain areas to be affected by any structural disconnection was spatially highly heterogenous. While in only 35% of patients the frontal pole was involved, all subjects showed at least some structural disconnection in the insular and precentral cortices as well as the subcortical regions of the caudate, pallidum, and putamen. Mixed effects logistic regression models did not show a significant difference between treatment groups for the propensity of brain regions to be affected by any degree of disconnection (Supplementary Table 2).

The median magnitude of non-zero ChaCo scores was also variable across brain regions, ranging from 1.43 × 10^−05^ (IQR [4.74 × 10^−06^, 4.85 × 10^−05^]) in the cuneus to 0.085 (IQR [0.019, 0.246]) in the putamen. After applying a logarithmic transformation, visual inspection of their distributions did not indicate a substantial deviation from the assumptions of conditional normality and constant variance (Supplementary Fig. 4). For brain-wide parametric analyses, positive ChaCo values were therefore modeled linearly on the logarithmic scale with a Gaussian response function.

**Fig. 2:**
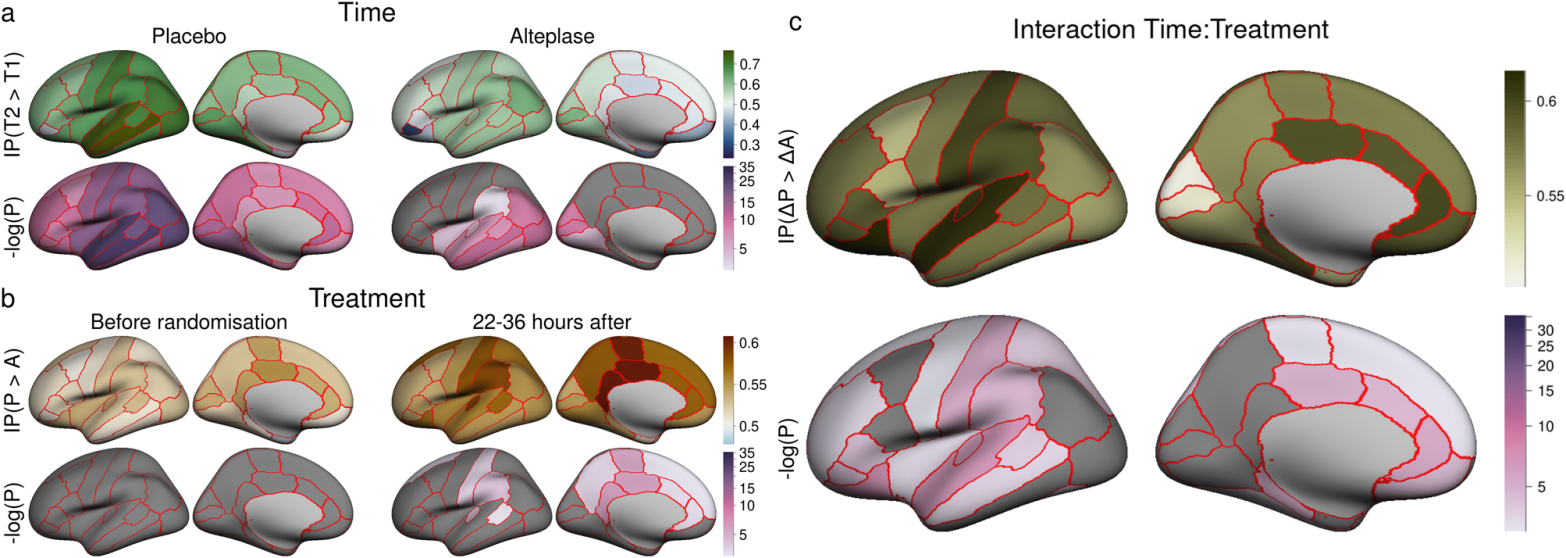
Non-parametric mass univariate analysis of effects of time and treatment allocation on loss of connectivity. In each panel, measures of effect size (top) and surprisal^51,62^, i.e. s = −log(P), (bottom) for each cortical parcel of the Desikan–Killiany atlas, are visualized as colored regions on the FreeSurfer standard template brain. The logarithmic scale for surprisal is identical across panels. Dark grey patches on surface representations correspond to uncorrected *P* values > 0.05. For corresponding numerical data see Supplementary Table 3. **a** Effect of time is quantified as the estimated probability ℙ(T2 > T1) that network disruption scores are higher at T2 than at T1 and assessed using Wilcoxon signed-rank tests. **b** Effect of treatment allocation is quantified as the estimated probability ℙ(P > A) that network disruption scores are higher in the placebo than in the alteplase group and assessed using Mann–Whitney U tests. **c** Interaction between time and treatment allocation is quantified as the estimated probability ℙ(ΔP > ΔA) that within-subject change (T2–T1) of network disruption scores is higher in the placebo than in the alteplase group and assessed using Mann–Whitney U tests. All *P* values are two sided.

Figure 2a visualizes the change in structural disconnection in individual cortical regions from before randomization to 22–36 h after stroke. In the placebo group, loss of connectivity was most likely in the temporal lobe, the insular, pre- and postcentral cortices, inferior and superior parietal cortices as well as in subcortical areas (thalamus, caudate nucleus, putamen). In the alteplase group, substantial loss of connectivity appeared more restricted, occurring mostly in the temporal lobe, pre- and postcentral and inferior temporal gyri. Differences in structural disconnection between treatment arms are represented in Fig. 2b. Before randomization, there were no significant differences in structural disconnection in any region (Mann–Whitney U P > 0.05). After 22–36 h, we observed an excess of disconnection in the placebo group compared to the alteplase group in the cingulum, para-, postcentral and supramarginal cortices, in the adjacent areas of the superiorfrontal and transverse temporal gyri as well as the precuneus and the caudate nucleus (uncorrected two-sided Wilcoxon P < 0.05). Interaction effects between time and treatment on structural disconnection are displayed in Fig. 2c. Numerically, within-subject change in disconnection in the placebo group was most likely to exceed that in the alteplase group in the transverse und superior temporal, postcentral, inferior frontal, and supramarginal gyri, the anterior cingulum as well as caudate nucleus. Detailed numerical results are reported in Supplementary Table 3.

**Fig. 3:**
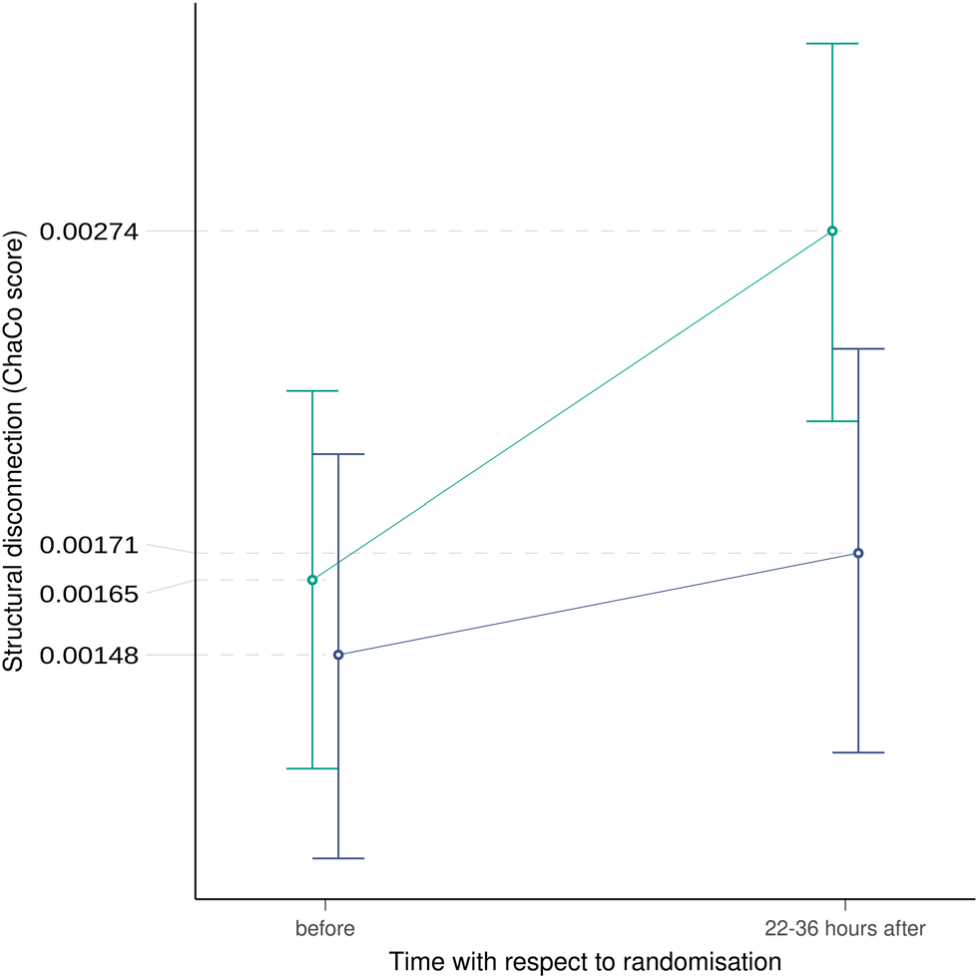
Progression of average structural disconnection after stroke from before (T1) through 22–36 h after (T2) randomization (*n* = 269 biologically independent subjects examined at two time points), stratified by allocation to placebo (persian green) or alteplase (tory blue). Circles represent structural disconnection quantified by mean average logarithmic Change of Connectivity (ChaCo) scores for the two groups. Vertical bars indicate 95% confidence intervals. The increase in structural disconnection, i.e. progressive loss of connectivity, is statistically significantly higher in the placebo group (*P* = 7.40 × 10^−4^ after adjustment for lesion volume and spatial heterogeneity, see also Table 1 for corresponding values).

After these descriptive analyses, we considered the profile of ChaCo scores across all pre-specified brain regions to assess structural disconnection at a global scale. Parametric modeling revealed a statistically significant interaction between time and treatment on global structural disconnection with more pronounced changes in the placebo group (Placebo: ChaCo^T2^/ChaCo^T1^ = 1.67, CI_95%_ [1.49, 1.88]; Alteplase: ChaCo^T2^/ChaCo^T1^ = 1.16, CI95% [1.02, 1.32]; *P* = 7.40 × 10^−4^; Table 1, Fig. 3). There was no evidence of spatial heterogeneity of this interaction effect. Effects of time and treatment allocation on connectivity, both for individual brain areas and pooled parametrically, were confirmed in an analogous analysis using the AAL atlas (Supplementary Tables 4, 5 and Supplementary Figs. 5–7).

**Table. 1:** Effect of time (T1 before and T2 after randomization) and treatment (placebo or alteplase) on global score of structural disconnection factoring in the effect of potential spatial heterogeneity in investigated brain areas. Shown is an ANOVA table for the linear-mixed effects model log(ChaCo+) ~ time × treatment + ROI × (time + log(volume)) + (1|subject), where the last term represents a random intercept for each subject. Two-sided *P* values are calculated from type II Wald χ^2^ tests, as implemented in the R package car^25^.

	χ²	d.o.f.	*P* value
**Main effects**			
Time	38.0	1	7.12e−10
Treatment	0.055	1	0.815
Time**:** Treatment	11.4	1	**7**.**40e−4**
**Covariates**			
log(Volume)	2133	1	<1e−300
**Spatial heterogeneity**			
ROI	27717	41	<1e−300
ROI**:** Time	71.6	41	0.002
ROI: log(Volume)	1468	41	<1e−300

*d.o.f.* degrees of freedom, *ROI* region of interest

### Progressive network disruption in combination with infarct growth, but not infarct growth alone, mediated the beneficial effect of thrombolysis

As shown above, treatment with alteplase was associated with better clinical outcome and less infarct growth. Also, infarct growth was strongly associated with unfavorable clinical outcome. In a causal mediation analysis adjusted for age, baseline NIHSS score, and pre-randomization DWI lesion volume, however, the effect of alteplase on the proportion of patients achieving a modified Rankin scale score of 0 or 1 after 3 months (favorable outcome) was not significantly mediated by lesion growth (natural indirect effect 1.04, CI_95%_ [0.96, 1.17], proportion mediated 8.3%, CI_95%_ [−8.0, 32.6]%, *P* = 0.193).

We next investigated whether the clinical effect of thrombolysis was better explained by considering change in structural connectivity as an additional mediator. As expected, this change in connectivity was not independent of lesion growth, with higher infarct growth rates associated with more pronounced loss of connectivity in all brain regions except the frontal and temporal poles (Supplementary Fig. 8). The associations between lesion growth and connectivity loss were strongest in the pre- and postcentral and supramarginal cortices, the insula, and the caudate, reflecting our selection of anterior-circulation stroke patients.

**Fig. 4:**
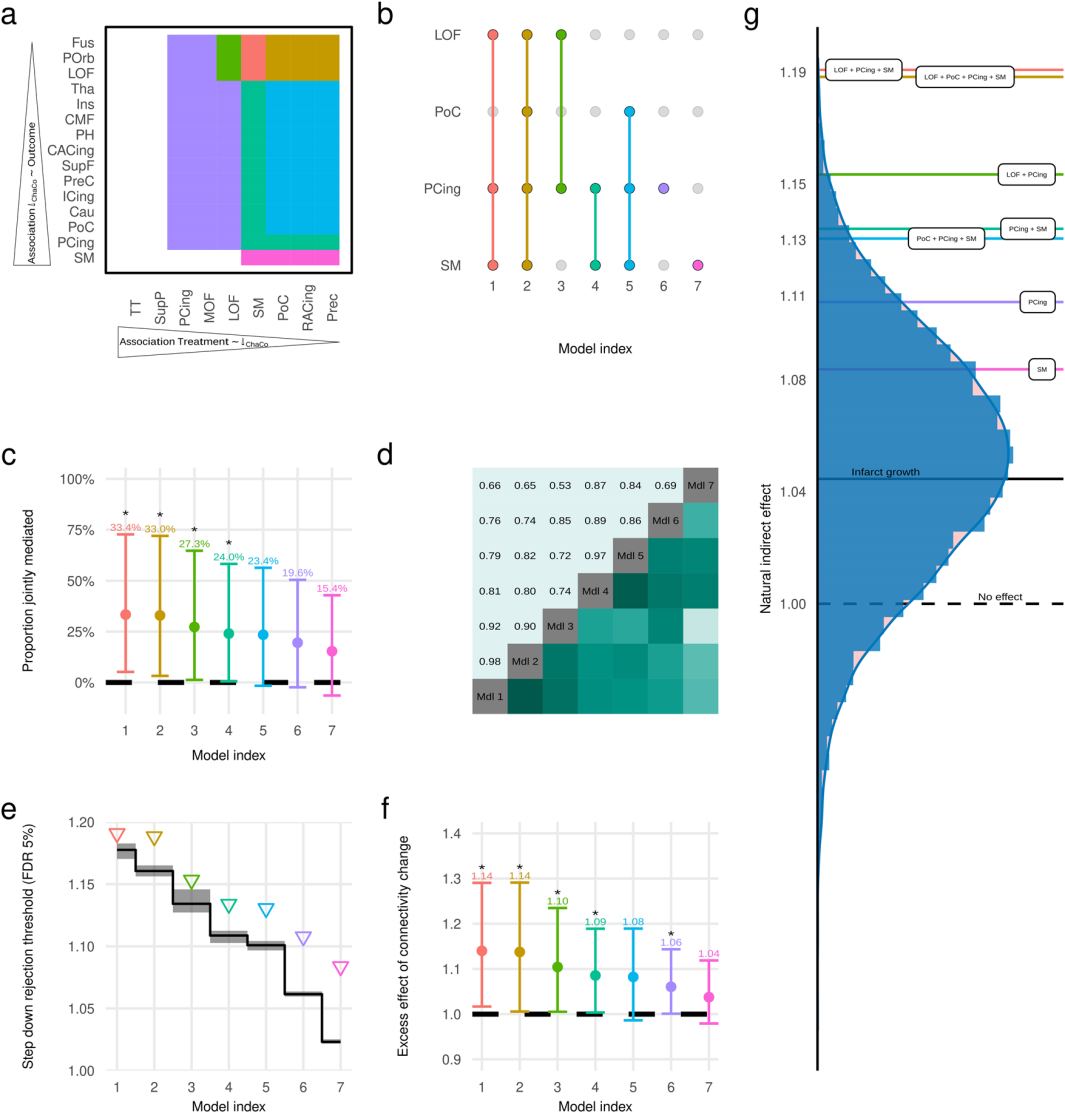
Results of multiple mediation analysis, adjusted for age, baseline NIHSS score and pre-randomization lesion volume. **a** Selection of brain regions by decreasing strength of association between treatment allocation and connectivity change (horizontal axis) and connectivity change and functional outcome (vertical axis). Each colored patch corresponds to a mediation model including, as mediators, infarct growth as well as connectivity change in the intersection of the *i* most alteplase-responsive (horizontal axis) and *j* most clinically eloquent (vertical axis) regions. Identical color coding is applied for the following subgraphs (**b**, **c**, **e**–**g**) to identify underlying referred models. **b** Composition of resulting seven mediation models comprising connectivity change in up to four brain regions. **c** Proportion of total effect of thrombolysis on favorable clinical outcome (mRS 0–1) mediated jointly through infarct growth and connectivity change in different sets of brain regions (*n* = 263 independent subjects). Solid dots represent point estimates. Vertical bars indicate 95% bias-corrected confidence intervals obtained from bootstrap with *k* = 10,000 replicates, stars (*) highlight models in which the 95%-CI does not contain 0%. **d** Empirical correlation between natural indirect effect estimates corresponding to different mediation models. Darker shades of green correspond to stronger correlations as indicated numerically in the left upper triangle of the matrix. **e** Step-down procedure to control the false discovery rate in the joint assessment of multiple mediation models at 5%. Models are ordered by their empirical natural indirect effect, indicated by triangles; beginning with the strongest effect, null hypotheses of no mediation effect are sequentially rejected as long as NIEs exceed their respective rejection threshold indicated by the black step line (median of *k* = 100 bootstrap replicates). Shading indicates 95% confidence intervals for the rejection line. **f** Point estimates (solid dots)and 95% confidence intervals (vertical bars) for the ratio between joint natural indirect effects of infarct growth and connectivity loss and the NIE of infarct growth alone (bootstrap with k = 10,000 replicates; *n* = 263 independent subjects). **g** Null distribution of empirical natural indirect effects mediated jointly through infarct growth and connectivity change in random subsets of all brain regions. Horizontal lines indicate the mediation effects of infarct growth alone (black, NIE 1.04) and infarct growth in combination with connectivity change in alteplase-responsive clinically eloquent areas (colored). NIE Natural indirect effect, SM supramarginal, PCing posterior cingulate, PoC postcentral, Cau caudate, ICing isthmus cingulate, PreC precentral, SupF superiorfrontal, CACing caudal anterior cingulate, PH parahippocampal, CMF caudal middle frontal, Ins insula, Tha thalamus, LOF lateral orbitofrontal, POrb pars orbitalis, Fus fusiform, TT transverse temporal, SupP superior parietal, MOF middle orbitofrontal, RACing rostral anterior cingulate.

In order to focus our analysis on brain regions with relevant connectivity alterations regarding treatment and clinical outcome, we first ranked brain regions in terms of their alteplase-responsiveness, i.e. association of connectivity change with treatment allocation (Table 2, left column), and clinical eloquence, i.e. association of connectivity change with favorable clinical outcome (mRS 0–1, Table 2, right column) using univariate linear and logistic regression analyses adjusted for age, baseline NIHSS score, and baseline lesion volume. We note that alteplase-responsiveness corresponds to the effect shown in Fig. 2c, where, however, no adjustment for covariates was performed. Overall, there was a high degree of similarity between both rankings (Spearman rank correlation 0.42, *P* 0.006). Connectivity changes in four brain regions showed significant associations with both treatment allocation and functional outcome as highlighted in Table 2 (supramarginal, postcentral, lateral orbitofrontal as well as the posterior cingulate cortex). These were explored as potential mediators of the effect of thrombolysis on functional outcome. Varying the thresholds for including a region according to either alteplase responsivity or clinical eloquence independently from very conservative (considering only the region with the strongest association) to very liberal (considering up to nine or fifteen regions, respectively) led to the identification of seven distinct models (Fig. 4a). These models contained the connectivity changes of up to four brain regions as mediators, their specific compositions are shown in Fig. 4b. The natural indirect effect of thrombolysis on functional outcome mediated jointly through infarct growth and connectivity loss in those sets of regions varied from 1.08 (CI_95%_ [0.97, 1.24]%) to 1.19 (CI_95%_ [1.03, 1.41]%), corresponding to proportions mediated between 15.4% (CI_95%_ [−3.9, 45.7]%) and 33.4% (CI_95%_ [8.8, 77.4]%) (Fig. 4c). As expected, mediation effects in these seven models were not independent, with bootstrap estimates of the correlation between natural indirect effects ranging from 0.53 to 0.98 (Fig. 4d). Step-down rejection thresholds for the null hypotheses of no mediation effect derived from the joint distribution of resampled indirect effects are shown in Fig. 4e. All seven observed indirect effects exceeded their respective threshold, thus providing evidence in favor of significant mediation in all models considered, while controlling the false discovery rate at 5%.

**Table 2:** Selection of brain regions for multiple mediation analysis.

The associations of change in connectivity from before to 22–36 h after randomization with treatment allocation (left) and favorable outcome (mRS 0–1 after 3 months, right) were assessed using linear and logistic regressions, respectively. Age, baseline NIHSS score and baseline lesion volume were included as covariates. Effect sizes were quantified by standardized regression coefficients (Beta) and standardized odds ratios (OR). Regions were ranked independently by the statistical evidence for a non-vanishing association between connectivity change and treatment/outcome and considered a potential mediator of the effect of thrombolysis if neither of the two 95% confidence intervals was compatible with the absence of an association (horizontal black lines). Matching regions are connected by lines to indicate similarity of the two rankings. Colors indicate assignment to frontal (cerulean), parietal (sun), temporal (dark cyan), occipital (summer sky) and limbic (violet red) lobes, or subcortical structures (orange).

**Fig. 5:**
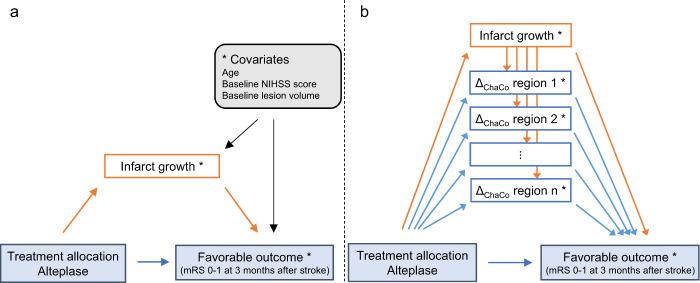
Diagrammatic representation of the causal models used in mediation analyses of the effect of thrombolysis with alteplase on functional outcome through structural parameters of infarct progression. Favorable clinical outcome was defined as mRS 0–1, 3 months after stroke. **a** Mediation by single mediator infarct growth. **b** Mediation by progressive loss of local structural connectivity (Δ_ChaCo_) in multiple brain regions. As a post-treatment confounder of the relation between change in connectivity and functional outcome, infarct growth was included as an additional mediator. Adjustment for covariates (age, lesion volume and NIHSS score at baseline prior to randomization) was carried out as marked by the asterisk and indicated explicitly in model a. mRS modified Ranking Scale; NIHSS National Institute of Health Stroke Scale.

A direct comparison of the models including only lesion growth (Fig. 5a) and the combination of lesion growth and connectivity changes (Fig. 5b) showed that the natural indirect effect mediated jointly through infarct growth and connectivity loss exceeded the NIE mediated through infarct growth alone by a factor of between 1.04 (CI_95%_ [0.98, 1.12]%) and 1.14 (CI_95%_ [1.02, 1.29]%) (Fig. 4f). For five of the seven models under consideration, this excess effect of connectivity change was statistically significant, as indicated by the bootstrap 95% confidence interval of the ratio of NIEs not containing 1. Connectivity change in brain regions that were not preselected by association with both treatment and outcome did not have, on average, an excess mediating effect as shown by the distribution of corresponding joint indirect effects centering around the indirect effect of infarct growth alone (Fig. 4g).

In an analogous analysis based on the Automatic Anatomical Labelling (AAL) atlas, results were qualitatively similar, albeit with somewhat smaller effect sizes that failed to reach statistical significance (Supplementary Fig. 9). Specifically, the selection of brain regions based on the association between their connectivity change and both treatment allocation and functional outcome identified three mediation models comprising of combinations of inferior parietal and middle cingulate cortices. In conjunction with infarct growth, connectivity change in these regions mediated between 12.4% (CI [−5.5, 39.4]%) and 16.1% (CI_95%_ [−5.5, 44.5]%) of the total clinical effect of thrombolysis. An additional model, corresponding to the best performing Model 1 in Fig. 4, was specified manually and included, as mediators, the inferior orbitofrontal, supramarginal, and angular cortices, as well as the cingulum. Together with infarct growth, local connectivity loss in these regions mediated 27.6% (CI_95%_ [−2.3, 64.9]%) of the total effect of thrombolysis on functional outcome. The combined natural indirect effect exceeded the NIE of infarct growth alone by a factor of 1.11 (CI_95%_ [0.96, 1.25]).

## Discussion

In this secondary analysis of structural connectome disruption in patients with anterior-circulation stroke from the WAKE-UP trial, we obtained two major results. Firstly, treatment with alteplase impeded growth of the ischemic lesion as well as stroke-induced loss of connectivity measured 22 to 36 h after stroke. Progression of structural disconnection was attenuated in clinically eloquent brain areas involved in motor control and speech. Secondly, averted loss of connectivity, in conjunction with prevented infarct growth, explained up to 33% of the total effect of alteplase on favorable clinical outcome measured by the modified Rankin scale 90 days after stroke. We observed a mediating effect of prevented connectivity loss on favorable outcome beyond the effect of lesion growth alone.

Consistent with the primary analysis of the WAKE-UP trial, stroke volumes in this subgroup of patients were small and did not differ between groups (placebo and alteplase) prior to randomization. In contrast to the main results of WAKE-UP, where neither final lesion stroke volumes nor lesion growth were significantly different between patients receiving alteplase or placebo^13^, we observed reduced lesion growth in the thrombolysis group. This disparity can be attributed to exclusion of patients with posterior-circulation and infratentorial stroke, where the growth of ischemic lesions is usually less extensive. Compared to previous large clinical trials, lesion volumes in WAKE-UP were determined from MRI (DWI) images, allowing a more accurate segmentation of infarcts compared to non-contrast CT and thus contributing to a higher sensitivity for detecting group differences in the evolution of lesion volume. The selection of patients with anterior-circulation stroke resulted in a lesion pattern focussed on structures in the deep territory of the middle cerebral artery, predominantly involving white matter structures and the subcortical grey matter (Supplementary Fig. 2).

A reduction of lesion size after randomization was observed in 24.5% of patients (66/269), matching previously reported rates of reversible DWI lesions in acute ischemic stroke^22^, which may indicate salvage of brain tissue. The association between DWI reversal and true reversal of cerebral ischemia is, however, confounded by several pathophysiological and imaging factors, and can vary with time to treatment, severity of perfusion deficits, lesion size, and site of vascular occlusion^22^.

In accordance with this lesion distribution, structural disconnections induced by stroke lesions were most pronounced in the basal ganglia as well as cortical areas of the frontal, parietal and temporal lobes adjacent to the central sulcus. We observed prominent disconnections of cortical areas such as the pre- and postcentral gyrus which are linked to subcortical lesion sites by long white matter fiber bundles such as the corticospinal or thalamo-cortical tract^23^. The observed anatomical pattern of connectome disruption corresponds well with previous observations in a group of 41 patients with acute ischemic stroke analyzed by a similar methodological approach using the Network Modification (NeMo) tool^24^. In this previous work, disconnection of brain areas involved in motor control and language was also associated with increases in the NIHSS score, indicating a relation between the pattern of structural disconnection and severity of clinical dysfunction^24^.

In our group of patients, a loss of global structural connectivity was observed over the first 22 to 36 h after stroke, manifesting as increasing Change of Connectivity (ChaCo) scores across nearly all brain areas. Growth of DWI lesions would be expected to lead to larger disruptions of the structural connectome. Similarly, lesion reduction can be associated with an increase in connectivity of specific brain areas. Biologically, thusly measured increased connectivity does not necessarily indicate de-novo growth of neurons at or through the site of ischemic injury but could rather represent the recovery of signal conduction along axonal projections running through salvageable brain tissue. Cytotoxic edema surrounding the ischemic core is likely to contribute to fluctuations of clinical deficits in stroke patients;^25,26^ we speculate that similarly, on a cellular level, reversal of cytotoxic enema as a consequence of reperfusion could lead to improved signal transduction along non-irreversibly damaged neurons via reduced physical pressure and the restoration of cellular metabolism^27,28^. However, given the unresolved question of an optimal ADC threshold to define the irreversibly damaged ischemic core, this pathophysiological interpretation of increased connectivity in the context of lesion size reduction has to remain speculative.

Our findings demonstrate that progression of structural disconnection was less extensive in patients treated with alteplase than in those receiving placebo. Notably, this effect of thrombolysis on preserved connectivity remained significant in a model adjusted for lesion volumes. This indicates that measures of structural disconnection contain information on the impact of stroke lesions and treatment effect of alteplase on brain structure that carry additional value compared to information gained by measuring lesion volumes alone. It emphasizes the significance of the anatomical pattern of infarction and is consistent with studies of lesion-symptom inference in stroke^14,29^. On a local scale, treatment-dependent attenuation of structural disconnections was numerically strongest in functionally eloquent areas adjacent to, but not directly affected by, the lesion, such as the inferior frontal, postcentral, supramarginal, and superior temporal gyrus (Fig. 2). However, these findings have to be interpreted with caution since there was no statistically significant heterogeneity.

Systemic thrombolysis is strongly linked to reperfusion, preservation of brain tissue, and favorable clinical outcome. However, currently used imaging biomarkers, such as infarct volume, are often poor surrogates for clinical outcome because they ignore the location-specific impact of a lesion on brain networks. Treatment with alteplase has been shown to impede the growth of the ischemic lesion, and to be associated with smaller final infarct volumes due to the restoration of blood flow in previously occluded arteries^30^. However, attenuation of lesion growth can be absent despite clinical treatment effects, even in patients with larger lesion volumes, after successful recanalization of large vessel occlusion^12^. In our subgroup analysis of the WAKE-UP trial, stroke lesion growth was reduced after alteplase treatment and associated with poor clinical outcome. Yet, in a causal mediation analysis, lesion growth did not explain a significant proportion of the clinical effect of alteplase on good outcome. While not statistically significant, the point estimate of the proportion mediated, 8.3%, is consistent with a recent study analyzing the mediation effect of final infarct volume on functional outcome in a larger cohort of acute stroke patients treated with endovascular therapy^31^. We could demonstrate that by considering averted loss of connectivity as additional structural mediators, up to a third of the total effect of thrombolysis on clinical outcome could be explained. The observed effect is in line with previous findings demonstrating higher clinical specificity of structural disconnection compared to purely focal measures of ischemic damage in stroke^20^. This finding in our patients is clinically plausible given the spatial pattern of brain regions in which loss of connectivity was responsive to alteplase treatment, (Fig. 4) and the use of the modified Ranking scale as a clinical outcome score in WAKE-UP. Specifically, our mediation models identified pronounced attenuation of disconnection in cortical areas involved in sensorimotor and language functions such as the postcentral cortex and inferior parietal lobule which would translate into better recovery measured by the mRS. In addition, averted connectivity loss in the posterior cingulum, a strategic and highly connected brain region involved in attention and various emotional and cognitive functions, featured prominently in all relevant mediation models demonstrating the surplus effect beyond lesion growth alone. Connectivity changes in brain areas at the center of the stroke lesion distribution in this cohort, such as the basal ganglia, insular or precentral gyrus did not show a large treatment response (Table 2), and were therefore not considered as mediators of the clinical effect of thrombolysis on favorable clinical outcome. While these findings are plausible in suggesting that the connectivity-preserving effect of thrombolysis might be most clinically relevant in connections supported in the ischemic penumbra around the infarct core, such an interpretation should be investigated further in future studies including more heterogeneous populations and alternative clinical outcome measures to determine which brain regions, viewed from a network perspective, are most sensitive to treatment effects and most critical for recovery.

In our study, we have chosen the mRS as a measure of functional outcome after stroke analogous to the primary endpoint of the WAKE-UP trial. Although the mRS is almost universally applied as an outcome measure in large clinical stroke trials, its validity in measuring functional outcome of stroke patients has been debated^32^. Specifically, the mRS broadly assesses the degree of disability or dependence in the daily activities, which strongly depend on intact motor functions. We speculate that our approach of detecting treatment-responsive areas of the structural connectome might also yield interesting insights into brain functions that rely on more extensive and complex brain networks, such as language, cognitive or affective functions relevant in stroke recovery^21^. Therefore, future studies using alternative outcome measures are needed.

Our results demonstrate the relevance of preserving structural connections in acute stroke treatment and offers a promising alternative to predict clinical recovery beyond conventional MRI imaging markers such as ischemic lesion volume. The strengths of our study are the use of well-defined stroke lesion segmentations based on the identification of ischemic tissue from MRI DWI within the framework of a large multicentre prospective clinical stroke trial. We furthermore apply an innovative and intuitive approach to estimate disturbances in structural brain networks that has previously been shown to be relevant for predicting clinical behavior and recovery in stroke populations^21,24^. Lastly, our statistical approach included adjustments for multiple parameters affecting clinical recovery, such as initial lesion volume and lesion growth. Although the current methodological approach still involves some manual data postprocessing and user interaction, we envision a more automated, time-efficient workflow for estimating structural disconnection from clinical stroke imaging data. Given the availability of automated lesion segmentation software in acute stroke diagnostic, an interesting approach would be to amend these tools with pre-installed structural connectomes that estimate the global or local disruptions in structural connectivity and its clinical impact depending on the choice of treatment and expected pattern of lesion growth based on the cerebral vascular architecture. Furthermore, we believe that future models and software applications based on automated classification algorithms to predict stroke outcome should take into account structural network changes as a relevant imaging marker in acute stroke.

In this study, structural connectivity damage was estimated indirectly by embedding stroke lesions masks in connectomes of healthy subjects. We have previously measured structural connectivity changes directly based on DTI and fiber tracking in a smaller group of acute stroke patients and reported a pattern of structural disconnection closely resembling our current findings^19^. However, substantial confounders exist when directly measuring acute stroke patients using the necessary high-resolution structural MRI protocols, such as excessive head movement artifacts specifically occurring in patients with more severe clinical deficits. There is also a potential selection bias towards more mildly affected patients who are better able to tolerate comparatively long examination times mandatory for robust measurement of signals in diffusion imaging. Collecting large datasets of stroke patients covering a broad range of lesion distributions and clinical deficits to achieve generalizable results is an additional challenge. Given these limitations, indirect estimation of disturbed structural connectivity in stroke is a valuable alternative for understanding the impact of focal lesion on long-range anatomical disconnections in relation to clinical behavior. In line with this hypothesis, several previous studies have successfully related indirect measures of disconnection to clinical phenotypes in stroke and small vessel disease^21,33–35^. Griffis et al. recently reported a mediation effect of indirect approximations of structural disconnection on empirically measured changes of resting-state functional connectivity in stroke patients, thus providing support for the functional relevance of indirectly assessed disconnectivity patterns^20^. The NeMo Tool used in our analysis has been shown to generate valid markers of disconnection that can help identify brain areas at risk of tissue loss after stroke and predict 6-months post-stroke outcome in various domains^21,36^. Associations between indirect connectivity measures (“disconnectomes”) and neuropsychological performances were also reported in patients with focal brain lesions of various etiologies^34^. Of note, analysis of reference data from different age groups (20 to 60 years) did not reveal a significant impact of age on resulting measures of structural disconnectivity. Notwithstanding, the marker of disconnectivity change employed in this study (ChaCo scores) has to be considered as a proxy of pathological neuronal disconnection contingent on the validity of the underlying reference connectome reconstruction methodology. Specifically, DTI tractography, by inferring connectivity from local diffusion orientation fields, is less reliable in brain areas with complex axonal fiber bundle configuration such as crossing and fanning fibers^37^. This effectively results in the generation of false-positive (erroneous) fiber tracts, whereas missing detections of valid fiber bundles (false-positive results) are less commonly observed^38^. Some disambiguation of complex fiber tract configurations can be achieved by technical improvements in diffusion tensor estimation such as High Angular Resolution Diffusion Imaging (HARDI), which was applied in our current analysis^39,40^. In terms of indirect estimations of structural connectivity changes as applied in our study, the aforementioned limitations would mainly translate to false-positive signals for brain areas with invalid association to the underlying stroke lesions. Although we have not noted a substantial amount of implausible disconnection in brain regions with anatomically unlikely connectivity to the observed lesion pattern (such as posterior brain areas, Fig. 1, Supplemental Figs. 4–6), we cannot exclude a methodological bias from DTI tractography affecting our results.

Moreover, by exploiting knowledge about the average anatomy of white matter tracts encoded in normative tractograms to extrapolate subject-specific lesion masks to expected changes in neural connections between grey matter areas, indirect lesion network mapping reflects the effects of individual focal lesions on a normative structural brain network^39^. It is therefore agnostic to inter-individual variability in white matter anatomy and integrity. The validity of our approach is thus contingent on the plausible assumption that, on average, inter-individual differences are, on average, much smaller than lesion-induced network disruptions. Given these limitations, there is a further need for direct comparison of direct and indirect measures of altered structural connectivity in relation to clinical phenotypes.

There are several additional limitations to our study that have to be considered. Firstly, the subset of the WAKE-UP trial cohort analyzed in this study comprised patients with anterior-circulation stroke, for whom follow-up DWI and FLAIR imaging after 22–36 h of sufficient quality for lesion segmentation was available. Clinical characteristics of excluded patients were similar to the study population, and therefore the risk of significant selection bias seems low. While it would be interesting to investigate the pattern of disconnection and its response to thrombolytic treatment in posterior-circulation strokes, the small number of such lesions in the WAKE-UP trial and the expected heterogeneity of connectivity profiles across lesion sites (specifically infratentorial strokes) led us to exclude these patients from analysis. Secondly, while the connectivity-preserving effect of thrombolysis could be demonstrated robustly in both the Desikan–Killiany and the AAL parcellation-based analysis, mediation effects of preserved connectivity could be reproduced only numerically in the AAL-based analysis but failed to reach statistical significance there. We believe this to be due to the higher spatial specificity of the Desikan atlas, which is derived from FreeSurfer’s surface-based cortical parcellation stream, whereas the AAL atlas is primarily volumetric, and tends to be more limited in its resolution of the intragyral grey-white matter interface. Thirdly, we are confident that our mediation model Fig. 5b is correctly specified; in the absence of a robust sensitivity analysis framework for models with multiple mediators, however, we cannot exclude the presence of unmeasured confounders of the relationship between connectivity loss and functional outcome^41,42^. Lastly, concerning the population of our study, patients were randomized in the WAKE-UP trial using an imaging paradigm that identifies patients whose stroke had likely occurred within the preceding 4.5 h, and who thus presented in a therapeutic time window for thrombolysis^43^. Future studies are needed to investigate the effect of earlier or later onset-to-treatment time intervals regarding trajectories of structural disconnection and outcome.

In conclusion, this post-hoc analysis of the WAKE-UP trial data provides evidence that systemic thrombolysis prevents progressive loss of structural connectivity after ischemic stroke, and that the associated preservation of the structural connectome contributes to mediating the clinical effect of thrombolytic treatment on functional outcome. Thus, while thrombolysis-induced reperfusion prevents tissue damage as measured by both lesion volume and loss of connectivity, the latter appears to be of independent relevance for clinical outcome. When evaluating the benefit of reperfusion therapy, structural connectivity may therefore provide an additional and perhaps more specific neuroimaging marker of treatment response beyond stroke lesion volume.

## Methods

### Study design

In this post-hoc analysis, we reviewed imaging and clinical data of all patients randomized in the WAKE-UP trial^13^. The WAKE-UP trial was an international, double-blind, placebo-controlled randomized clinical trial that studied the efficacy and safety of systemic thrombolysis with alteplase in acute ischemic stroke patients with unknown time of symptom onset. Patients were included after MR imaging had demonstrated an acute ischemic lesion in diffusion-weighted sequences without established FLAIR hyperintensity. This mismatch had previously been shown to indicate that the stroke most likely occurred within 4.5 h from imaging^43^. The WAKE-UP trial demonstrated that thrombolysis in patients with unknown symptom onset is effective and safe if this imaging criterion is fulfilled.

For our secondary analysis, we included patients with isolated anterior-circulation stroke who received diffusion-weighted imaging (DWI) and fluid-attenuated inversion recovery (FLAIR) imaging at two pre-specified time points of the WAKE-UP trial. We included data from initial MRI examinations prior to randomization (T1) and follow-up imaging 22 to 36 h after randomization (T2). Due to the small number of patients with posterior-circulation infarcts and the substantial heterogeneity of structural network disturbance between patients with anterior- and posterior-circulation lesions, we restricted our analysis to patients with stroke in the anterior circulation. We excluded data from patients with bilateral stroke, as well as imaging data of insufficient quality, i.e. those exhibiting large motion artifacts, preventing correct segmentation and registration of stroke lesions. Written informed consent by either patients or their legal representatives was provided according to relevant regulations. The WAKE-UP trial was approved by Ethics Committee of the Hamburg Chamber of Physicians, the German Federal Institute for Drugs and Medical Devices, as well as by local competent authorities and ethics committees at each study site.

### Imaging data processing and analysis

Imaging data were analyzed using a dedicated software developed for the WAKE-UP trial (Stroke Quantification Tool, SONIA, v1.0) based on previous methods and functionalities of a stroke imaging toolbox developed in-house^44,45^. In summary, individual DWI and FLAIR datasets were coregistered using rigid transformation. Maps of apparent diffusion coefficient (ADC) were calculated based on two DWI datasets chosen automatically with b-values of 0 s/mm² and between 500 s/mm² and 1500 s/mm² according to the imaging protocol of the individual study site. Stroke lesions were segmented on ADC maps using a semi-automated procedure with initial manual delineation and secondary automated refinement based on an ADC threshold of 620 mm²/s. All images were visually checked for quality and plausibility of segmentation results by cross-modal inspection of FLAIR and ADC maps. All segmentations were reviewed by two neurologists experienced in acute stroke treatment and stroke imaging research (BC and AK) and were reviewed a second time by a board-certified neurologist (BC) and manually corrected, if necessary. Lesion volumes were calculated based on final lesion segmentations in native space. Binary lesion masks were transformed to MNI space by applying linear and non-linear registrations. All lesions were visually checked after transformation to MNI space and corrected if necessary.

### Quantification of structural network disruption

Binary lesion masks were entered into the Network Modification (NeMo) toolbox^39^ for Matlab^46^ to estimate structural disconnection at the level of individual grey-matter regions, defined in the Desikan–Killiany FSL^47^ and Automated Anatomical Labelling (AAL)^48^ atlas with 86 and 116 regions, respectively. In summary, the NeMo tool quantifies disconnections in the brain’s structural network by mapping stroke lesions from clinical imaging data onto a collection of connectomes from 73 healthy participants. The NeMo tool has been applied to investigate lesion-dysfunction relationships^24^, white- and grey-matter pathologies in normal aging^49^, as well as prediction of 6-month clinical outcome after stroke^21^. Specifically, for each brain area and each of 73 whole-brain reference tractograms provided in the NeMo toolbox, the number of streamlines connected to this region and running through the site of the lesion was counted and divided by the total connectivity of the region. The average of these 73 proportions, referred to as the Change of Connectivity (ChaCo) score, was computed as a measure of local structural disconnection. In this score, values of zero indicate that the streamlines originating from a grey-matter area do not pass through the stroke region at all (no disconnection), while the maximal value one represents complete disconnection of a piece of cortex from the rest of the network. In our analysis, ChaCo scores were computed for stroke hemispheres only, which, after exclusion of the cerebellar regions, were parcellated into 42 (Desikan–Killiany) or 45 (AAL) areas. Although our sample did not include patients with cerebellar stroke and the cerebellum was not investigated as a region of interest, we note that cerebellar streamlines contributing to the connectivity of other affected regions, such as the thalamus, were not discarded. We calculated ChaCo scores from imaging data both prior to and 22 to 36 h after randomization. Given that at both times, network disruption was computed from the same of reference tractograms, the difference in ChaCo scores was interpreted as the absolute within-subject loss of connectivity.

### Clinical data

We collected clinical data from the database of the WAKE-UP trial, including patient age at symptom onset, National Institutes of Health Stroke Scale (NIHSS) score at presentation and functional outcome 90 days after stroke. In addition, information on randomization (treatment with alteplase or placebo) was collected. For our analysis, we defined the clinical endpoint following the primary efficacy endpoint of the WAKE-UP trial as a score of 0 or 1 point on the modified Rankin Scale (mRS) of neurologic disability (ranging from 0 [no symptoms] to 6 [death]), assessed 90 days after stroke.

### Statistical analysis

Baseline characteristics, including age and NIHSS score at initial presentation, were compared between patients assigned to treatment with placebo and alteplase using a two-sample t-test and Mann–Whitney U test, respectively.

Growth of lesion volume between T1 (initial MRI prior to randomization) and T2 (MRI at 22 to 36 h after randomization) was assessed using Wilcoxon signed-rank tests^50^. A Mann–Whitney U test was performed to compare absolute within-subject lesion volume change between placebo and alteplase groups and assess the effect of treatment on infarct growth. In addition, generalized linear-mixed effects modeling was carried out to assess the interaction effect between treatment and time on lesion volume in a parametric setting. An unconditional logistic regression model, adjusted for age, baseline NIHSS score and baseline lesion volume, was fitted to associate the log odds of achieving an mRS score of 0-1 with infarct growth. The adjusted odds ratio was tested against the null hypothesis of no association using a Wald t-test and presented with a 95% confidence interval obtained from profiling the likelihood function.

For each brain area, the evolution of ChaCo scores over time, differences between placebo and alteplase groups, as well as the interaction between treatment and time, were explored non-parametrically by contrasting median connectivity scores within and between groups. For illustration we also report the Shannon information or surprisal as a measure of the information against the hypothesis of no effect encoded in the data^51^. While these are derived from Wilcoxon signed-rank and Mann–Whitney U tests, no formal null-hypothesis significance testing was intended and, consequently, no adjustment for multiple testing was performed. To assess our main hypothesis with regards to the global effects of time and treatment on ChaCo scores, linear-mixed effects regressions were fit to pooled data from all areas. In a two-component mixture model, the probability of non-zero ChaCo score was first analyzed using logistic regressions; positive ChaCo values were then transformed logarithmically and analyzed with a constant-variance Gaussian response distribution. Given the potential confounding effect of infarct size, all parametric models included logarithmically transformed lesion volume as a covariate.

Statistical analyses were performed in the R Statistical Computing Environment (version 4.0.2)^52^.

### Mediation analysis

To explain the mechanisms underlying the clinical benefit of systemic thrombolysis with alteplase, we performed a causal mediation analysis in the counterfactual framework^53^. We specified the hypothetical causal models represented in Fig. 5, in which the clinical effect of thrombolysis on functional outcome would be mediated either through lesion growth alone (a) or through the combination of lesion growth and structural connectivity decline in multiple brain areas (b). Infarct growth was included as a mediator in model (b) to account for its confounding effect on the relation between connectivity decline and functional outcome. Both models included the pre-treatment covariates age, baseline NIHSS and pre-randomization lesion volume. Given the inclusion of both baseline lesion volume and infarct growth in all models, final infarct volume was not explicitly included to avoid collinearity.

The strength of mediation was quantified using natural effects in the counterfactual framework^54^. Specifically, natural direct, natural indirect and total effects were calculated on the odds ratio scale as

1 $$\text{NDE}=\frac{{{P}}_{\text{T},\text{M|P}}/(1-{{P}}_{\text{T},\text{M|P}})}{{{P}}_{\text{P},\text{M|P}}/(1-{{P}}_{\text{P},\text{M|P}})},\quad\text{NIE}=\frac{{{P}}_{\text{T},\text{M|T}}/(1-{{P}}_{\text{T},\text{M|T}})}{{{P}}_{\text{T},\text{M|P}}/(1-{{P}}_{\text{T},\text{M|P}})},\quad\text{TE}=\text{NIE}\times \text{NDE},$$

where *P*_T, M|T_ and *P*_P, M|P_ are the expected probabilities of a favorable clinical outcome (mRS ≤ 1) after receiving thrombolysis or placebo, respectively^54^. The remaining term *P*_T, M|P_ is the expected counterfactual probability of a favorable clinical outcome for the thrombolysis group if the distribution of the mediator had been fixed to what it would have been under the placebo condition. The proportion jointly mediated through all mediators included in a model was quantified as the ratio log(NIE)/log(TE). All counterfactual probabilities were estimated using the inverse probability weighting approach, employing simple logistic regressions as exposure and outcome models. Weighting is a flexible approach to mediation applicable in the context of non-rare binary outcomes which allows for causal relations between different mediators and does not require estimation of separate regression models for each of the mediators^55^.

A prerequisite for mediation to occur is that the exposure affects the mediator and the mediator affects the outcome^56^. We therefore included regions as mediators in the model 5-b based on the strength of association between treatment allocation and local connectivity decline, and local connectivity decline and functional outcome, varying the threshold for both criteria independently.

Mediation models were assessed statistically using the simple bootstrap with *k* = 10,000 resamples. Uncertainty in estimated natural indirect effects and proportions mediated is reported as bias-corrected 95% confidence intervals. To prevent type I error inflation in the context of assessing more than one mediation model we chose to control the false discovery rate at 5%. Since we expected models with different, but overlapping sets of mediating brain regions to exhibit a high degree of statistical dependence, we used bootstrap samples to construct a sequence of rejection thresholds in a step-down procedure that takes into account the joint distribution of natural effects across models^57^. Uncertainty in the rejection thresholds was assessed by bootstrapping the bootstrapped NIE estimates (*k* = 100 resamples).

In the multiple mediation model (5-b), the natural indirect effect of thrombolysis mediated through prevented loss of connectivity, but not through prevented infarct growth, cannot be statistically identified^58^. We therefore compared models (5-a) and (5-b) using the same bootstrap approach as above and derived 95% confidence intervals for the difference between the natural indirect effect mediated jointly through prevented infarct growth and preserved connectivity on the one hand and prevented infarct growth alone on the other hand.

Finally, we investigated whether any mediation effect of preserved local connectivity would be specific to the alteplase-responsive clinically eloquent regions identified above. We thus sampled uniformly k = 10,000 sets of brain regions, estimated the corresponding mediation model (Fig. 5b) and constructed a null distribution of natural indirect effects. We also repeated all mediation analyses using the imputation-based approach implemented in the R package medflex v.0.6-6, which confirmed our results^59–61^.

### Reporting summary

Further information on research design is available in the Nature Research Reporting Summary linked to this article.

## Supplementary information

Supplementary Information

Reporting Summary

## Data Availability

All patient-level data used in the analysis, including age, baseline NIHSS score, lesion volumes, treatment allocation, connectivity measurements, and functional outcome, are available on GitHub (https://github.com/csi-hamburg/WAKE-UP-preserved-SC). Imaging data, after de-identification, will be shared with the Virtual International Stroke Trials Archive (VISTA) and be accessible according to the VISTA rules (http://www.virtualtrialsarchives.org/vista).

## Code availability

Code for all analyses presented in the paper and for the creation of figures is available on GitHub (https://github.com/csi-hamburg/WAKE-UP-preserved-SC).
