# Supplementary material for: Preserved structural connectivity mediates the clinical effect of thrombolysis in patients with anterior-circulation stroke
Source: Nat Commun. 2021 May 10;12:2590. doi: 10.1038/s41467-021-22786-w (PMC8110812; doi:10.1038/s41467-021-22786-w)
Supplement: Supplementary file 1 — Supplementary Information [file 41467_2021_22786_MOESM1_ESM.pdf]

SUPPLEMENTARY INFORMATION TO

PRESERVED STRUCTURAL CONNECTIVITY MEDIATES THE CLINICAL EFFECT OF THROMBOLYSIS IN PATIENTS WITH ANTERIOR-CIRCULATION STROKE

Eckhard Schlemm*[,1], Thies Ingwersen[1], Alina Königsberg[1], Florent Boutitie[2, 3, 4], Martin Ebinger[5, 6], Matthias Endres[5,7,8,9,10], Jochen B. Fiebach[5], Jens Fiehler[11], Ivana Galinovic[5], Robin Lemmens[12,13,14], Keith W. Muir[15], Norbert Nighoghossian[16], Salvador Pedraza[17], Josep Puig[17], Claus Z. Simonsen[18], Vincent Thijs[19,20], Anke Wouters[12,13,14], Christian Gerloff[1], Götz Thomalla[1], Bastian Cheng[1]

*Corresponding author email e.schlemm@uke.de

[1] Klinik und Poliklinik für Neurologie, Kopf- und Neurozentrum, University Medical Center Hamburg-Eppendorf, Martinistr. 52, 20246 Hamburg, Germany.

[2] Hospices Civils de Lyon, Service de Biostatistique, F-69003 Lyon, France

[3] Université Lyon 1, F-69100 Villeurbanne, France

[4] CNRS, UMR 5558, Laboratoire de Biométrie et Biologie Evolutive, Equipe Biostatistique-Santé, F-69100 Villeurbanne, France

[5] Centrum für Schlaganfallforschung Berlin (CSB), Charité - Universitätsmedizin Berlin, Campus Mitte, Charitéplatz 1, 10117 Berlin, Germany

[6] Klinik für Neurologie, Medical Park Berlin Humboldtmühle, An der Mühle 2-9, 13507 Berlin, Germany

[7] Klinik und Hochschulambulanz für Neurologie, Charité-Universitätsmedizin Berlin, Campus Mitte, Charitéplatz 1, 10117 Berlin, Germany

[8] German Center for Neurodegenerative Diseases (DZNE), partner site Berlin

[9] German Centre for Cardiovascular Research (DZHK), partner site Berlin

[10] ExcellenceCluster NeuroCure

[11] Department of Diagnostic and Interventional Neuroradiology, University Medical Center Hamburg-Eppendorf, Martinistr. 52, 20246 Hamburg, Germany

[12] Department of Neurology, University Hospitals Leuven, Herestraat 49, 3000 Leuven, Belgium

[13] KU Leuven – University of Leuven, Department of Neurosciences, Experimental Neurology, Oude Markt 13, bus 5005, 3000 Leuven, Belgium

[14] VIB, Center for Brain & Disease Research, Laboratory of Neurobiology, Campus Gasthuisberg, Herestraat 49, bus 602, 3000 Leuven, Belgium

[15] Institute of Neuroscience & Psychology, University of Glasgow, University Avenue, Glasgow G12 8QQ, UK

[16] Department of Stroke Medicine, Université Claude Bernard Lyon 1, CREATIS CNRS UMR 5220-INSERM U1206, INSA-Lyon; Hospices Civils de Lyon, Lyon, France

[17] Department of Radiology, Institut de Diagnostic per la Image (IDI), Hospital Dr Josep Trueta, Institut d'Investigació Biomèdica de Girona (IDIBGI), Parc Hospitalari Martí i Julià de Salt - Edifici M2, 17190 Salt, Girona, Spain

[18] Department of Neurology, Aarhus University Hospital, 8200 Aarhus, Denmark

[19] Stroke Division, Florey Institute of Neuroscience and Mental Health, University of Melbourne, 245 Burgundy Street, Heidelberg, VIC 3084, Victoria, Australia

[20] Austin Health, Department of Neurology, 145 Studley Road, Heidelberg, VIC 3084, Australia

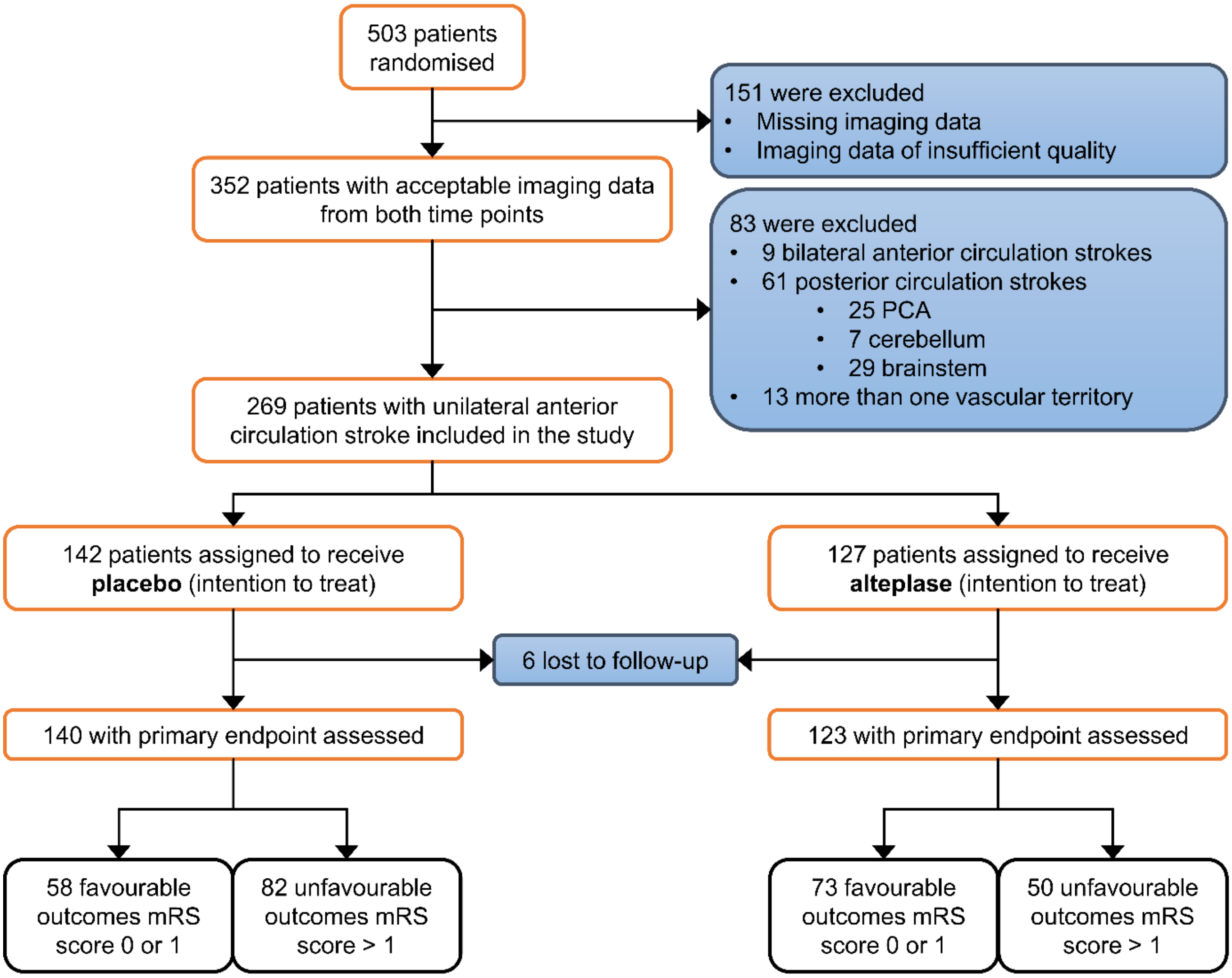

Supplementary Fig. 1: Flow chart detailing application of exclusion criteria, composition of the study population, treatment allocation, and clinical outcomes.

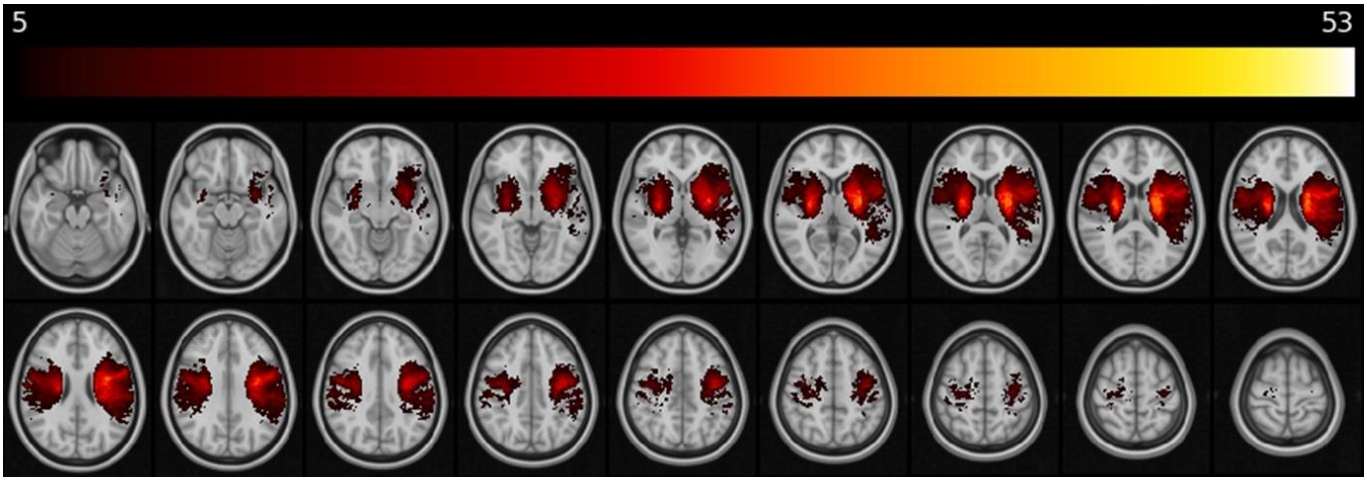

Supplementary Fig. 2: Overlay of 269 anterior-circulation stroke lesions included in the analysis. Stroke lesions have been segmented from pre-treatment diffusion-weighted MR imaging and registered to MNI152 space. Voxels that are affected by at least five infarcts are marked.

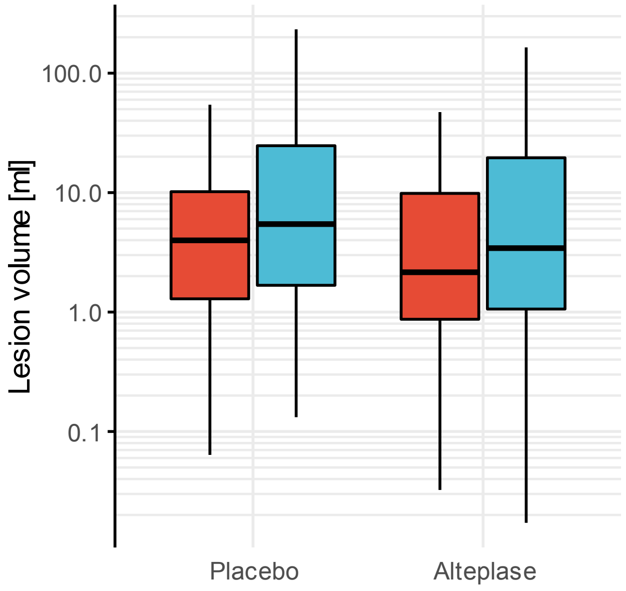

Supplementary Fig. 3: Infarct size on a logarithmic scale stratified by treatment allocation and time after stroke (n=269 biologically independent subjects examined at two time points). The volume increase from before (cinnabar) to 22 – 36 hours after randomization (turquoise) is significantly less in the alteplase group than in the placebo group (nonparametric two-sided P = 0.018). Boxes and horizontal bars indicate interquartile range (IQR, lower bound Q_1, upper bound Q_3) and median (Q_2), respectively. Whiskers extend to the minimum (Q_0) and maximum (Q_4) values of lesion volume. There are no outlier data greater than Q_3 + 1.5*IQR.

	Generalized Poisson			Negative binomial		
	Estimate	SE	P	Estimate	SE	P
**Model: Volume ~ Time × Treatment + (1|subject)**						
(Intercept)	1.415	0.132	6.99e-27	1.350	0.127	3.34e-26
$\text{Treatment}_{\text{Alteplase}}$	-0.142	0.179	0.429	-0.136	0.180	0.450
Time_{T2}	0.927	0.064	3.17e-48	0.964	0.055	1.03e-68
Time_{T2} : $\text{Treatment}_{\text{Alteplase}}$	-0.234	0.092	**0.011**	-0.239	0.083	**0.004**
**Model: $\text{Volume}^{\text{T2}}$ ~ Treatment + log($\text{Volume}^{\text{T1}}$)**						
(Intercept)	1.707	0.121	6.36e-45	1.739	0.117	3.64e-50
log($\text{Volume}^{\text{T1}}$)	0.728	0.040	7.90e-73	0.716	0.040	3.61e-71
$\text{Treatment}_{\text{Alteplase}}$	-0.179	0.088	**0.042**	-0.182	0.085	**0.031**

Supplementary Tab. 1: Regression tables including point estimates of regression coefficients, standard errors (SE), and two-sided P values for generalized mixed-effects regressions of lesion volume with generalized Poisson and negative binomial response distributions. Two-sided P values corresponding to the effect of systemic thrombolysis on the temporal evolution of lesion volume are marked in bold. Two-sided P values are calculated from the *z* ratios of the coefficients based on a normal reference distribution, as implemented in the R package glmmTMB.[1] Since models represent alternative approaches to analyzing the same effect, no adjustment multiple comparison is made.

	χ^2	d.o.f.	P
**Main effects**			
Time	17.0	1	3.68e-5
Treatment	0.91	1	0.341
Time **:** Treatment	2.22	1	0.136
**Covariates**			
log(Volume)	327	1	3.95e-73
**Spatial heterogeneity**			
ROI	12005	41	< 1e-300
ROI **:** Time	666	41	< 1e-300
ROI **:** log(Volume)	790	41	< 1e-300

Supplementary Tab. 2: ANOVA table for the generalized linear mixed-effects model $I_{\{ChaCo > 0\}}$ ~ time × treatment + ROI × (time + log(volume)) + (1|subject),), where the last term represents a random intercept for each subject. Two-sided P values are calculated from type II Wald χ^2 tests, as implemented in the R package car.[2]

d.o.f. = degrees of freedom. ROI = region of interest

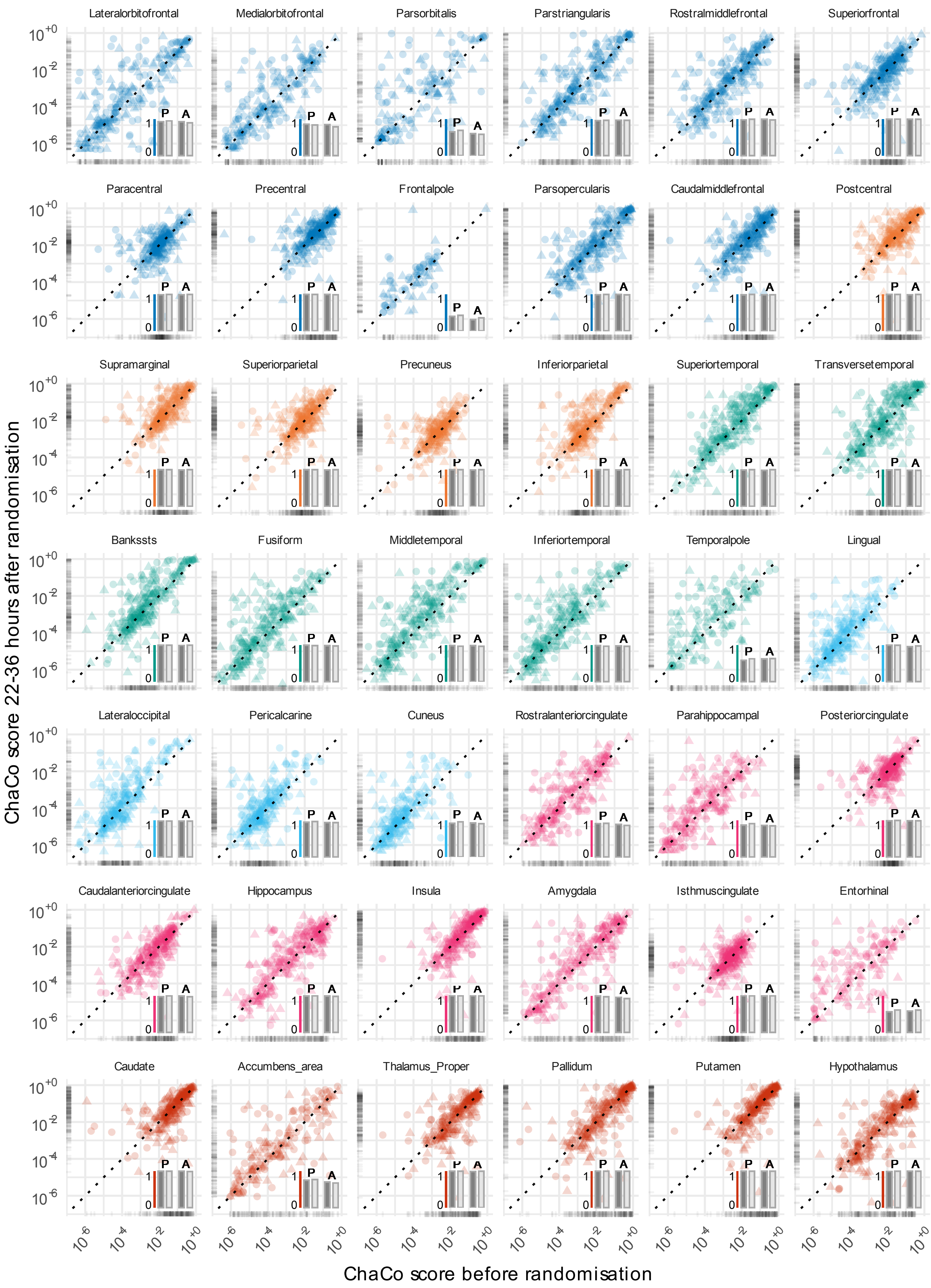

Supplementary Fig. 4: Distribution of positive ChaCo score within regions of interest of the Desikan—Killiany atlas before and 22 – 36 hours after randomization on a bi-logarithmic scale. Shape indicates treatment allocation for each individual subject (n=269) to either placebo (circle) or alteplase (triangle), color indicates anatomical assignment to frontal (cerulean), parietal (sun), temporal (dark cyan), occipital (summer sky) and limbic (violet red) lobes, or subcortical structures (orange). Inset bars represent the proportion of non-zero ChaCo scores before (light grey) and after (dark grey) randomization for the Placebo (P) and Alteplase (A) groups.

	Main effect of treatment*						Main effect of time†						Interaction‡	
	all		T1		T2		all		Placebo		Alteplase			
ROI	ES	P	ES	P	ES	P	ES	P	ES	P	ES	P	ES	P
Lateralorbitofrontal	0.553	**0.035**	0.536	0.308	0.569	0.050	0.550	**0.006**	0.641	**6.38e-05**	0.449	0.681	0.616	**0.001**
Superiortemporal	0.542	0.093	0.525	0.474	0.560	0.090	0.669	**9.38e-14**	0.746	**2.01e-12**	0.583	**0.001**	0.613	**0.001**
Transversetemporal	0.561	**0.015**	0.535	0.317	0.586	**0.015**	0.632	**6.33e-10**	0.690	**1.68e-09**	0.567	**0.016**	0.608	**0.002**
Rostralanteriorcingulate	0.556	**0.024**	0.538	0.283	0.574	**0.036**	0.543	**7.14e-04**	0.599	**1.56e-05**	0.480	0.793	0.601	**0.004**
Postcentral	0.553	**0.035**	0.527	0.449	0.580	**0.024**	0.643	**9.10e-08**	0.697	**2.12e-08**	0.583	0.075	0.600	**0.005**
Parahippocampal	0.526	0.291	0.508	0.829	0.545	0.204	0.550	**1.09e-04**	0.627	**2.99e-06**	0.465	0.559	0.599	**0.005**
Posteriorcingulate	0.582	**0.001**	0.553	0.134	0.607	**0.002**	0.532	0.056	0.592	**0.001**	0.465	0.523	0.595	**0.007**
Supramarginal	0.558	**0.020**	0.528	0.425	0.587	**0.014**	0.636	**2.24e-08**	0.676	**3.00e-08**	0.591	**0.037**	0.594	**0.008**
Caudalanteriorcingulate	0.558	**0.019**	0.540	0.252	0.576	**0.032**	0.554	**0.002**	0.620	**5.43e-05**	0.480	0.833	0.593	**0.009**
Medialorbitofrontal	0.528	0.256	0.508	0.827	0.547	0.181	0.480	**0.031**	0.528	**9.12e-04**	0.425	0.681	0.590	**0.011**
Parsorbitalis	0.524	0.323	0.508	0.813	0.541	0.236	0.387	**0.004**	0.465	**1.59e-04**	0.299	0.844	0.586	**0.013**
Parstriangularis	0.530	0.232	0.517	0.628	0.543	0.226	0.546	**0.004**	0.599	**2.74e-04**	0.488	0.724	0.586	**0.015**
Superiorparietal	0.539	0.114	0.512	0.730	0.566	0.061	0.613	**6.82e-07**	0.662	**1.61e-07**	0.559	0.089	0.583	**0.019**
Bankssts	0.551	**0.040**	0.531	0.386	0.572	**0.042**	0.639	**5.45e-12**	0.690	**8.15e-10**	0.583	**8.57e-04**	0.581	**0.021**
Caudate	0.561	**0.015**	0.548	0.179	0.576	**0.032**	0.569	**5.11e-04**	0.641	**5.92e-05**	0.488	0.467	0.581	**0.022**
Fusiform	0.530	0.229	0.517	0.622	0.542	0.237	0.636	**2.83e-09**	0.711	**1.03e-08**	0.551	**0.013**	0.580	**0.024**
Accumbens_area	0.536	0.146	0.515	0.670	0.556	0.108	0.424	0.111	0.486	**0.007**	0.354	0.509	0.580	**0.024**
Thalamus_Proper	0.539	0.121	0.513	0.714	0.564	0.071	0.554	**0.003**	0.627	**3.28e-04**	0.472	0.690	0.579	**0.026**
Middletemporal	0.529	0.245	0.510	0.781	0.548	0.171	0.684	**3.78e-16**	0.768	**6.34e-13**	0.591	**2.54e-05**	0.577	**0.029**
Hippocampus	0.520	0.417	0.494	0.872	0.546	0.191	0.584	**4.28e-04**	0.641	**5.58e-05**	0.520	0.406	0.576	**0.032**
Insula	0.545	0.069	0.536	0.304	0.556	0.112	0.617	**1.57e-08**	0.648	**5.86e-08**	0.583	**0.014**	0.576	**0.032**
Pallidum	0.533	0.184	0.515	0.675	0.552	0.139	0.543	**0.016**	0.599	**0.001**	0.480	0.949	0.576	**0.032**
Putamen	0.541	0.104	0.529	0.406	0.554	0.129	0.599	**2.66e-05**	0.655	**2.66e-05**	0.535	0.116	0.575	**0.033**
Rostralmiddlefrontal	0.524	0.329	0.513	0.708	0.536	0.310	0.569	**6.67e-05**	0.606	**2.12e-05**	0.528	0.193	0.574	**0.036**
Superiorfrontal	0.550	**0.046**	0.526	0.455	0.572	**0.042**	0.561	**0.002**	0.613	**2.67e-04**	0.504	0.506	0.572	**0.042**
Paracentral	0.576	**0.002**	0.544	0.211	0.604	**0.003**	0.554	**0.009**	0.613	**8.63e-04**	0.488	0.719	0.571	**0.043**

	Main effect of treatment[*]						Main effect of time[†]						Interaction[‡]	
	all		T1		T2		all		Placebo		Alteplase			
ROI	ES	P	ES	P	ES	P	ES	P	ES	P	ES	P	ES	P
Precentral	0.530	0.233	0.510	0.768	0.549	0.166	0.613	**1.52e-06**	0.655	**8.39e-07**	0.567	0.062	0.569	**0.049**
Amygdala	0.521	0.396	0.502	0.966	0.539	0.268	0.580	**7.70e-05**	0.641	**4.08e-05**	0.512	0.181	0.568	0.053
Hypothalamus	0.525	0.319	0.494	0.872	0.553	0.134	0.543	**0.024**	0.606	**0.003**	0.472	0.907	0.568	0.056
Isthmuscingulate	0.578	**0.002**	0.544	0.214	0.608	**0.002**	0.524	**0.015**	0.556	**0.002**	0.488	0.761	0.565	0.068
Precuneus	0.554	**0.032**	0.527	0.441	0.579	**0.026**	0.572	**9.40e-05**	0.606	**4.89e-05**	0.535	0.151	0.564	0.071
Inferiorparietal	0.536	0.148	0.521	0.545	0.551	0.149	0.662	**4.72e-13**	0.697	**2.85e-10**	0.622	**1.28e-04**	0.563	0.073
Lingual	0.538	0.125	0.520	0.577	0.557	0.106	0.617	**3.68e-08**	0.648	**2.82e-07**	0.583	**0.011**	0.560	0.089
Frontalpole	0.537	0.085	0.533	0.281	0.542	0.173	0.286	**0.001**	0.331	**9.80e-05**	0.236	0.760	0.560	0.063
Inferiortemporal	0.529	0.245	0.513	0.706	0.544	0.212	0.636	**2.21e-10**	0.683	**3.91e-08**	0.583	**8.74e-04**	0.559	0.093
Lateraloccipital	0.537	0.137	0.526	0.462	0.550	0.161	0.651	**4.47e-14**	0.704	**1.17e-10**	0.591	**3.38e-05**	0.557	0.109
Entorhinal	0.497	0.913	0.483	0.619	0.512	0.730	0.413	**1.09e-04**	0.451	**4.00e-04**	0.370	0.057	0.546	0.182
Parsopercularis	0.526	0.298	0.516	0.661	0.538	0.283	0.595	**3.83e-05**	0.627	**1.03e-04**	0.559	0.052	0.546	0.193
Caudalmiddlefrontal	0.520	0.425	0.505	0.881	0.535	0.319	0.569	**5.66e-04**	0.592	**9.22e-04**	0.543	0.131	0.542	0.240
Temporalpole	0.498	0.937	0.485	0.671	0.511	0.741	0.409	**0.002**	0.415	**0.007**	0.402	0.123	0.522	0.527
Pericalcarine	0.542	0.095	0.537	0.291	0.547	0.180	0.636	**1.06e-09**	0.655	**2.63e-06**	0.614	**1.20e-04**	0.516	0.656
Cuneus	0.538	0.128	0.536	0.307	0.540	0.259	0.584	**1.33e-08**	0.599	**1.84e-05**	0.567	**1.95e-04**	0.506	0.864

Supplementary Tab. 3: Statistical details of non-parametric mass-univariate analysis of the effects of treatment allocation and time on ChaCo score in regions of interest of the Desikan–Killiany atlas. Brain areas are sorted by effect size of the interaction between time and treatment allocation. All P values are two-sided.

[*] estimated probability that ChaCo in the Placebo group exceeds ChaCo in the Alteplase group; P value from Mann–Whitney U test

[†] estimated probability that ChaCo at T2 exceeds ChaCo at T1; P value from Wilcoxon signed-rank test

[‡] estimated probability that within-subject ChaCo difference (T2 minus T1) in the Placebo group exceeds within-subject ChaCo difference (T2 minus T1) in the Alteplase group; P value from Mann–Whitney U test

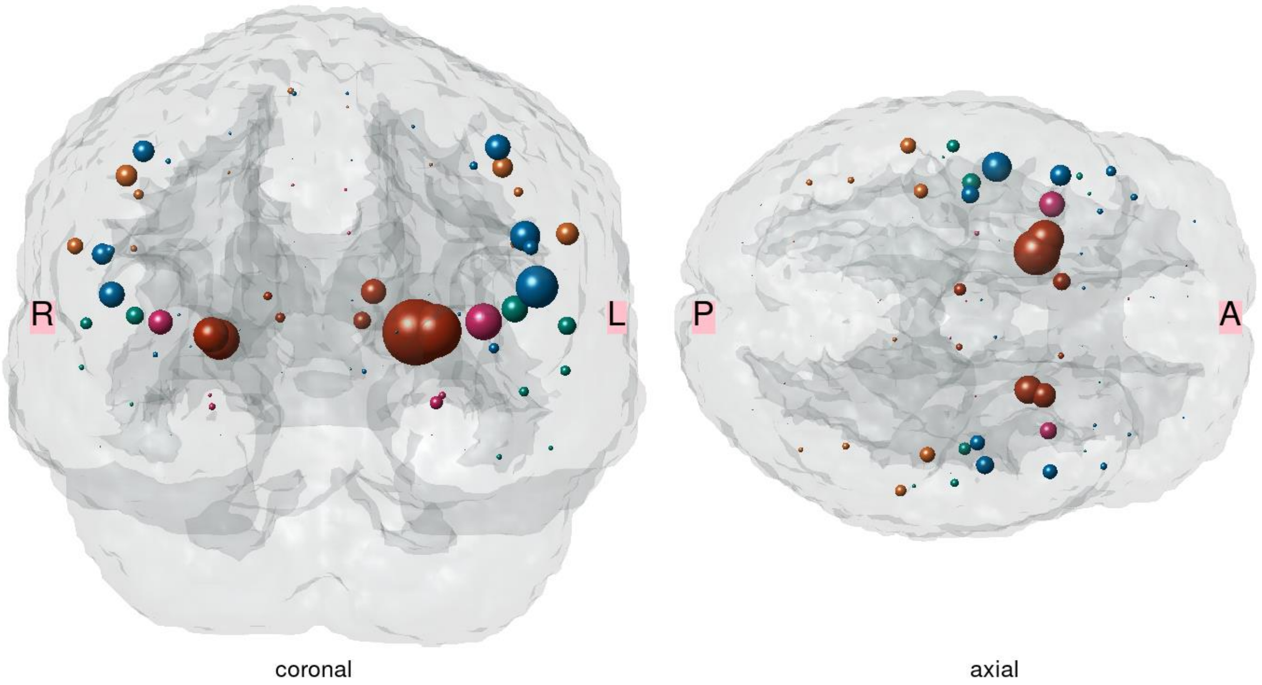

Supplementary Fig. 5: Structural network disruption induced by mapping ischemic stroke lesions segmented from pre-randomization diffusion imaging onto reference tractograms from 73 healthy subjects (NeMo toolbox). Spheres are located at the center of gravity of each of 2 × 58 brain regions of the AAL atlas. Volume indicates magnitude of average change of connectivity (n=269 subjects) with larger sizes indicating higher degree of disconnection. Colors indicate assignment to frontal (cerulean), parietal (sun), temporal (dark cyan), occipital (summer sky) and limbic (violet red) lobes, or subcortical structures (orange). This Figure was created using the NeMo toolbox.[3]

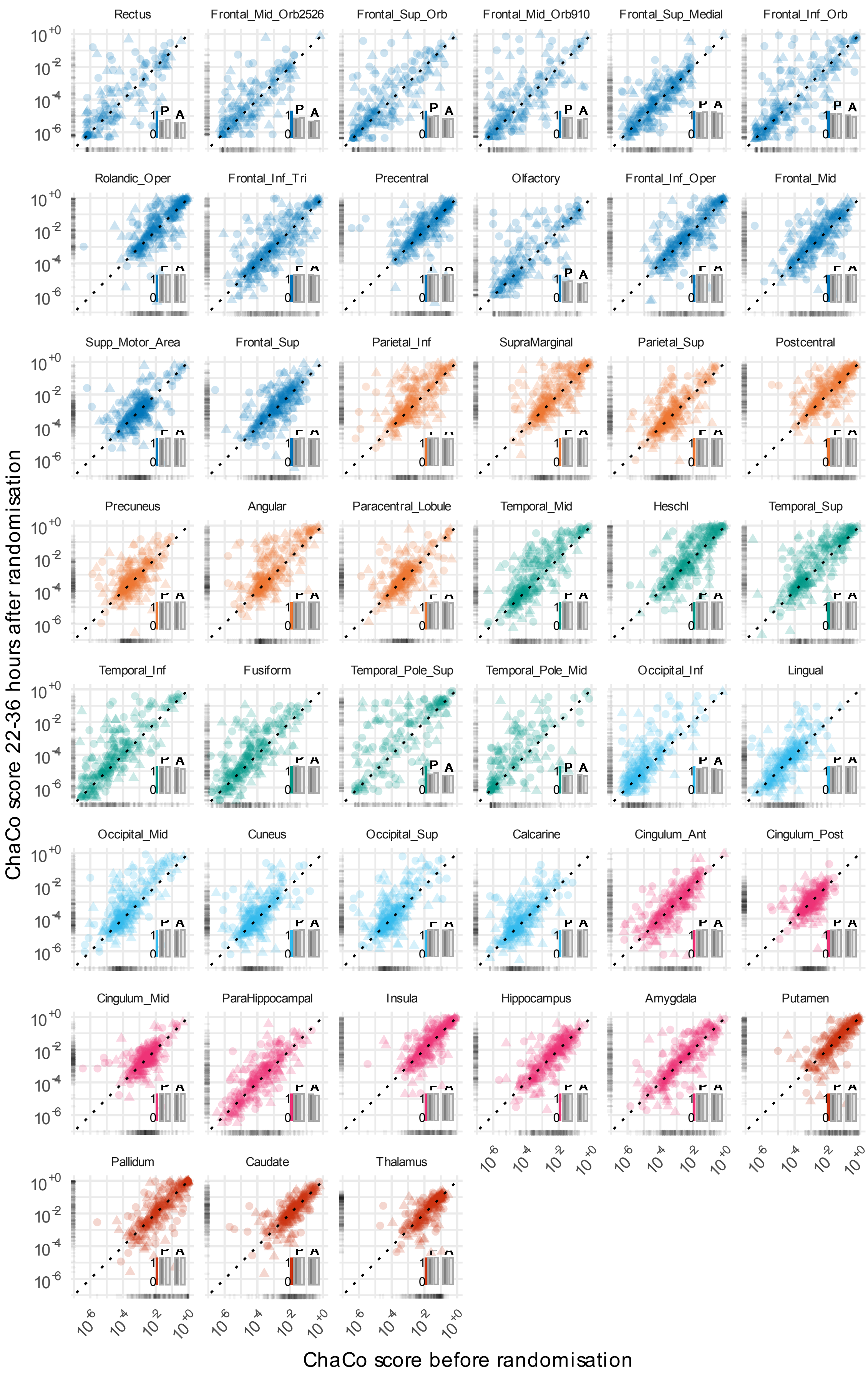

Supplementary Fig. 6: Distribution of positive ChaCo score within regions of interest of the AAL atlas before and 22 – 36 hours after randomization on a bi-logarithmic scale. Shape indicates treatment allocation for each individual subject (n=269) to either placebo (circle) or alteplase (triangle), color indicates anatomical assignment to frontal (cerulean), parietal (sun), temporal (dark cyan), occipital (summer sky) and limbic (violet red) lobes, or subcortical structures (orange). Inset bars represent the proportion of non-zero ChaCo scores before (light grey) and after (dark grey) randomization for the placebo (P) and alteplase (A) groups.

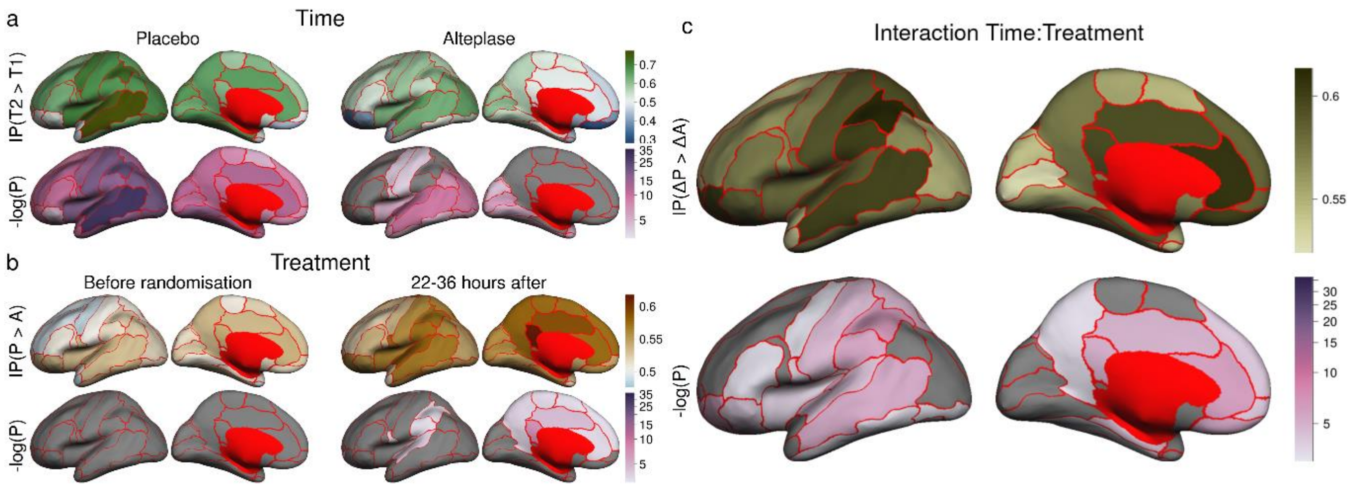

Supplementary Fig. 7: Non-parametric mass univariate analysis of effects of time and treatment allocation on loss of connectivity. In each panel, measures of effect size (top) and surprisal[4,5], i.e. s=-log(p), (bottom) for each cortical parcel of the AAL atlas, are visualized as colored regions on the Colin template brain.[6] The logarithmic scale for surprisal is identical across panels. Dark grey patches on surface representations correspond to uncorrected two-sided P values > 0.05. For corresponding numerical data see Supplementary Table 5. **a** Effect of time is quantified as the estimated probability that network disruption scores are higher at T2 than at T1 and assessed using Wilcoxon signed-rank tests. **b** Effect of treatment allocation is quantified as the estimated probability that network disruption scores are higher in the placebo than in the alteplase group and assessed using Mann-Whitney U tests. **c** Interaction between time and treatment allocation is quantified as the estimated probability that within-subject change (T2-T1) of network disruption scores is higher in the placebo than in the alteplase group and assessed using Mann-Whitney U tests. All P values are two-sided. This Figure was created using FreeSurfer.[7]

	Main effect of treatment*						Main effect of time†						Interaction‡	
	all		T1		T2		all		Placebo		Alteplase			
ROI	ES	P	ES	P	ES	P	ES	P	ES	P	ES	P	ES	P
Parietal_Inf	0.548	0.055	0.522	0.543	0.573	**0.039**	0.662	**2.62e-09**	0.718	**2.56e-10**	0.598	0.066	0.614	**0.001**
Cingulum_Ant	0.557	**0.023**	0.538	0.284	0.575	**0.034**	0.569	**4.91e-04**	0.648	**6.22e-06**	0.480	0.846	0.609	**0.002**
SupraMarginal	0.544	0.080	0.515	0.662	0.573	**0.039**	0.613	**5.55e-07**	0.669	**7.03e-08**	0.551	0.155	0.603	**0.004**
Frontal_Mid_Orb2526	0.546	0.058	0.533	0.342	0.560	0.082	0.428	**0.008**	0.514	**1.98e-04**	0.331	0.818	0.601	**0.004**
Temporal_Mid	0.549	**0.048**	0.531	0.384	0.569	0.051	0.706	**1.36e-16**	0.775	**3.75e-14**	0.630	**7.98e-05**	0.601	**0.004**
Rectus	0.536	0.133	0.516	0.646	0.556	0.101	0.372	**0.034**	0.437	**4.96e-04**	0.299	0.467	0.599	**0.004**
Heschl	0.569	**0.005**	0.545	0.198	0.593	**0.009**	0.621	**3.08e-09**	0.662	**1.32e-08**	0.575	**0.015**	0.597	**0.006**
Cingulum_Post	0.580	**0.001**	0.540	0.258	0.618	**8.62e-04**	0.565	**0.001**	0.613	**5.43e-05**	0.512	0.776	0.596	**0.007**
Frontal_Sup_Orb	0.549	**0.046**	0.529	0.402	0.569	**0.049**	0.450	**0.016**	0.521	**4.01e-04**	0.370	0.676	0.596	**0.006**
Cingulum_Mid	0.562	**0.014**	0.533	0.346	0.589	**0.012**	0.584	**1.76e-04**	0.655	**4.56e-06**	0.504	0.545	0.595	**0.007**
Parietal_Sup	0.534	0.173	0.505	0.883	0.561	0.084	0.613	**3.88e-06**	0.655	**2.31e-07**	0.567	0.196	0.593	**0.009**
Postcentral	0.532	0.205	0.503	0.923	0.560	0.090	0.636	**3.96e-09**	0.676	**3.78e-09**	0.591	**0.023**	0.592	**0.009**
Temporal_Sup	0.558	**0.021**	0.535	0.319	0.580	**0.023**	0.703	**4.71e-16**	0.746	**2.85e-12**	0.654	**1.40e-05**	0.588	**0.012**
Putamen	0.538	0.125	0.522	0.535	0.556	0.116	0.572	**9.47e-04**	0.641	**8.92e-05**	0.496	0.582	0.587	**0.014**
Pallidum	0.531	0.221	0.514	0.683	0.548	0.174	0.561	**0.012**	0.620	**6.33e-04**	0.496	0.984	0.585	**0.016**
ParaHippocampal	0.534	0.177	0.508	0.811	0.559	0.095	0.587	**7.34e-06**	0.662	**9.02e-07**	0.504	0.192	0.585	**0.016**
Frontal_Mid_Orb910	0.533	0.181	0.523	0.513	0.543	0.215	0.439	**0.029**	0.486	**0.001**	0.386	0.726	0.584	**0.016**
Frontal_Sup_Medial	0.552	**0.039**	0.531	0.386	0.573	**0.039**	0.524	**0.003**	0.585	**2.37e-04**	0.457	0.744	0.584	**0.017**
Frontal_Inf_Orb	0.546	0.064	0.534	0.329	0.558	0.097	0.461	0.189	0.521	**0.010**	0.394	0.377	0.583	**0.019**
Caudate	0.560	**0.016**	0.547	0.181	0.574	**0.037**	0.546	**0.019**	0.606	**0.001**	0.480	0.989	0.582	**0.021**
Insula	0.550	**0.045**	0.532	0.360	0.566	0.060	0.658	**1.41e-09**	0.690	**1.27e-08**	0.622	**0.006**	0.580	**0.024**
Rolandic_Oper	0.554	**0.032**	0.530	0.401	0.574	**0.035**	0.628	**3.89e-08**	0.662	**1.07e-07**	0.591	**0.022**	0.579	**0.025**
Precuneus	0.557	**0.023**	0.534	0.337	0.578	**0.027**	0.606	**5.73e-05**	0.648	**2.38e-05**	0.559	0.183	0.573	**0.038**
Frontal_Inf_Tri	0.521	0.407	0.506	0.865	0.536	0.306	0.580	**1.02e-04**	0.627	**3.83e-05**	0.528	0.198	0.572	**0.042**
Temporal_Inf	0.546	0.065	0.534	0.335	0.558	0.099	0.591	**1.31e-08**	0.655	**8.30e-08**	0.520	**0.011**	0.571	**0.045**
Occipital_Inf	0.535	0.160	0.516	0.647	0.554	0.128	0.625	**1.01e-10**	0.690	**3.40e-09**	0.551	**0.002**	0.570	**0.047**
Precentral	0.510	0.683	0.487	0.706	0.534	0.340	0.647	**7.75e-08**	0.697	**2.36e-07**	0.591	**0.020**	0.570	**0.049**

	Main effect of treatment*						Main effect of time†						Interaction‡	
	all		T1		T2		all		Placebo		Alteplase			
ROI	ES	P	ES	P	ES	P	ES	P	ES	P	ES	P	ES	P
Fusiform	0.542	0.090	0.530	0.398	0.555	0.120	0.628	**8.65e-09**	0.683	**4.65e-08**	0.567	**0.011**	0.569	**0.050**
Thalamus	0.540	0.106	0.516	0.655	0.565	0.064	0.580	**5.67e-04**	0.655	**6.29e-05**	0.496	0.379	0.567	0.056
Olfactory	0.532	0.195	0.517	0.634	0.546	0.186	0.480	**0.002**	0.528	**6.81e-04**	0.425	0.406	0.566	0.059
Frontal_Inf_Oper	0.522	0.385	0.512	0.745	0.533	0.350	0.587	**1.42e-04**	0.634	**5.29e-05**	0.535	0.191	0.566	0.063
Angular	0.541	0.099	0.523	0.513	0.560	0.089	0.647	**5.08e-11**	0.669	**5.40e-09**	0.622	**7.92e-04**	0.565	0.067
Frontal_Mid	0.506	0.814	0.489	0.759	0.522	0.538	0.599	**4.57e-06**	0.641	**6.10e-06**	0.551	0.057	0.561	0.084
Hippocampus	0.543	0.085	0.520	0.567	0.566	0.061	0.558	**4.27e-04**	0.613	**4.04e-04**	0.496	0.206	0.557	0.105
Paracentral_Lobule	0.543	0.086	0.507	0.852	0.575	**0.034**	0.554	**7.37e-04**	0.585	**5.20e-04**	0.520	0.222	0.554	0.128
Lingual	0.530	0.231	0.509	0.807	0.551	0.148	0.610	**2.48e-08**	0.662	**6.33e-07**	0.551	**0.006**	0.553	0.134
Amygdala	0.537	0.140	0.518	0.617	0.556	0.115	0.580	**2.19e-04**	0.641	**3.57e-04**	0.512	0.139	0.550	0.161
Occipital_Mid	0.531	0.212	0.517	0.627	0.544	0.215	0.662	**5.23e-12**	0.676	**3.22e-09**	0.646	**1.52e-04**	0.548	0.179
Supp_Motor_Area	0.548	0.055	0.521	0.556	0.573	**0.039**	0.569	**0.009**	0.613	**0.006**	0.520	0.417	0.547	0.187
Temporal_Pole_Sup	0.521	0.388	0.509	0.789	0.533	0.339	0.483	**3.97e-08**	0.528	**2.28e-06**	0.433	**0.003**	0.546	0.186
Frontal_Sup	0.509	0.708	0.487	0.716	0.531	0.379	0.591	**1.33e-04**	0.606	**1.73e-04**	0.575	0.098	0.546	0.197
Cuneus	0.538	0.127	0.515	0.668	0.557	0.106	0.632	**4.02e-07**	0.662	**5.70e-06**	0.598	**0.011**	0.543	0.222
Occipital_Sup	0.537	0.137	0.515	0.661	0.555	0.121	0.636	**1.55e-07**	0.655	**3.33e-06**	0.614	**0.007**	0.543	0.225
Temporal_Pole_Mid	0.499	0.970	0.482	0.594	0.516	0.648	0.435	**2.76e-07**	0.451	**1.30e-05**	0.417	**0.003**	0.536	0.293
Calcarine	0.541	0.101	0.523	0.507	0.558	0.100	0.617	**2.37e-07**	0.634	**2.37e-05**	0.598	**0.002**	0.527	0.438

Supplementary Tab. 4: Statistical details of non-parametric mass-univariate analysis of the effects of treatment allocation and time on ChaCo score in regions of interest of the AAL atlas. Brain areas are sorted by effect size of the interaction between time and treatment allocation. All P values are two-sided.

* estimated probability that ChaCo in the Placebo group exceeds ChaCo in the Alteplase group; P value from Mann–Whitney U test

† estimated probability that ChaCo at T2 exceeds ChaCo at T1; P value from Wilcoxon signed-rank test

‡ estimated probability that within-subject ChaCo difference (T2 minus T1) in the Placebo group exceeds within-subject ChaCo difference (T2 minus T1) in the Alteplase group; P value from Mann–Whitney U test

	log(ChaCo⁺)~ time × treatment + ROI × (time + vol) + (1∣subject)			I(ChaCo > 0) ~ time × treatment + ROI × (time + vol) + (1∣subject)		
	χ^2	d.o.f.	P value	χ^2	d.o.f.	P
**Main effects**						
Time	16.0	1	6.25e-05	16.7	1	4.44e-5
Treatment	0.11	1	0.735	0.013	1	0.910
Time: Treatment	12.1	1	**5.14e-04**	3.23	1	0.072
**Covariates**						
log(Volume)	2415	1	< 1e-300	445	1	9.28e-99
**Spatial heterogeneity**						
ROI	26127	44	< 1e-300	7520	44	< 1e-300
ROI : Time	80.8	44	6.07e-04	239	44	1.28e-28
ROI : log(Volume)	1427	44	< 1e-300	422	44	3052e-63

Supplementary Tab. 5: ANOVA tables for the two-component mixture model relating ChaCo score in the regions of the AAL atlas to treatment allocation and time, adjusting for lesion volume and spatial heterogeneity. Two-sided P values are calculated from type II Wald χ^2 tests, as implemented in the R package car,[2] and from the *z* ratios of the coefficients based on a normal reference distribution, as implemented in the R package glmmTMB.[1]

d.o.f. = degrees of freedom. ROI = region of interest

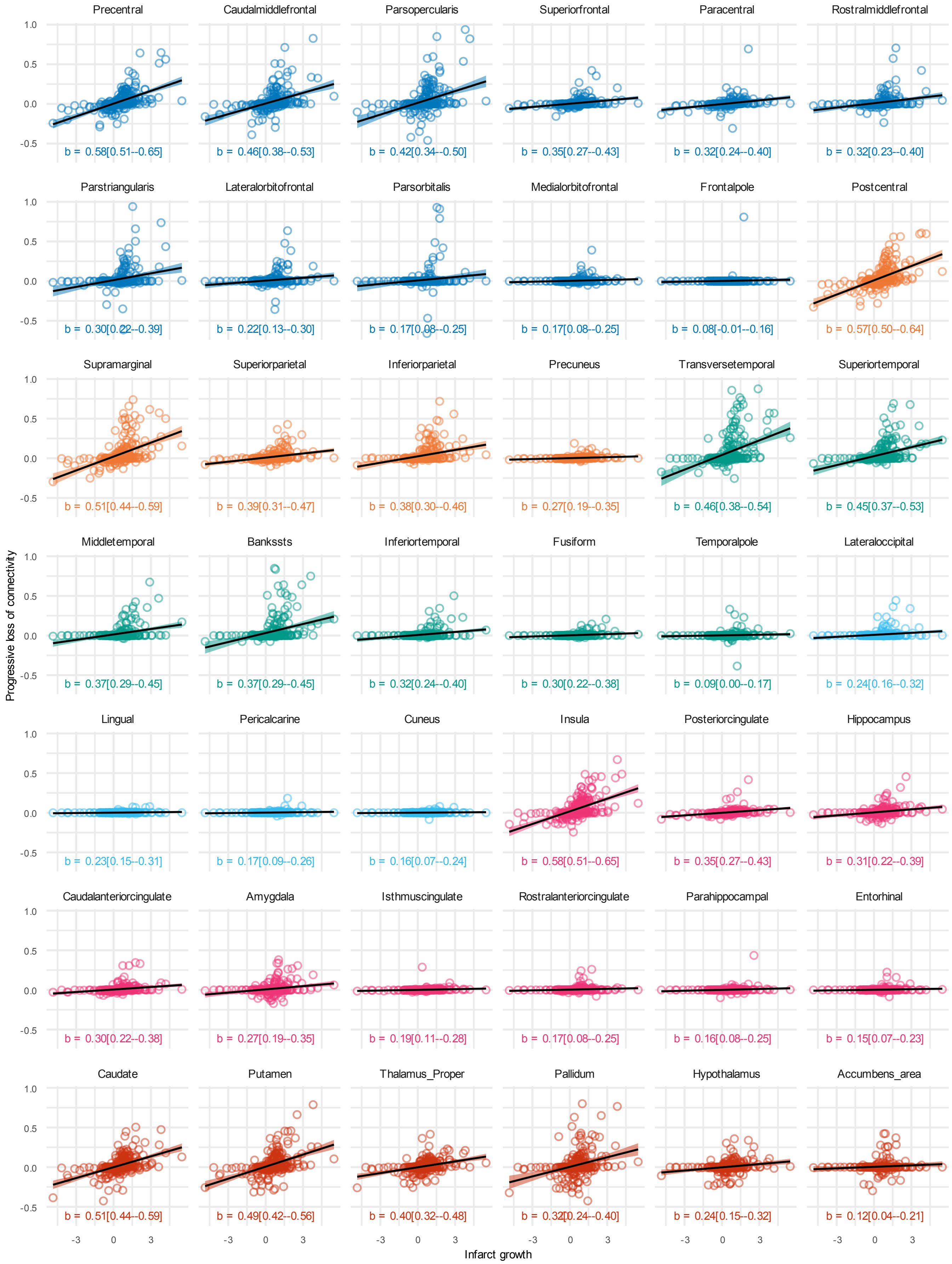

Supplementary Fig. 8. Linear association between progressive loss of structural connectivity (Δ_{ChaCo}) in regions of the Desikan—Killiany parcellation and infarct growth (log(lesionvolume^{T2}) - log(lesionvolume^{T1})). Black lines represent point estimates of population means of progressive loss of connectivity; shaded areas represent corresponding pointwise 95% confidence intervals. Text insets report standardised unadjusted regression coefficients *b* and 95% confidence intervals. Colours indicate assignment to frontal (cerulean), parietal (sun), temporal (dark cyan), occipital (summer sky) and limbic (violet red) lobes, or subcortical structures (orange). Results for the AAL atlas were similar.

Association Δ_{ChaCo} ~Treatment

Beta	95% CI	Region
-0.04	[-0.06, -0.01]	Cingulum_Post
-0.05	[-0.09, -0.02]	Parietal_Inf
-0.01	[-0.01, 0.00]	Parietal_Sup
-0.03	[-0.05, 0.00]	Cingulum_Mid
0.00	[0.00, 0.00]	Rectus
-0.01	[-0.02, 0.00]	Precuneus
0.00	[0.00, 0.00]	Frontal_Sup_Orb
-0.01	[-0.02, 0.00]	Cingulum_Ant
-0.09	[-0.18, 0.00]	Putamen
0.00	[0.00, 0.00]	Frontal_Mid_Orb910
0.00	[-0.01, 0.00]	Paracentral_Lobule
0.00	[0.00, 0.00]	Frontal_Inf_Orb
-0.08	[-0.17, 0.01]	Heschl
-0.06	[-0.12, 0.01]	Caudate
-0.04	[-0.09, 0.01]	SupraMarginal
-0.09	[-0.20, 0.01]	Postcentral
-0.02	[-0.04, 0.00]	Temporal_Mid
0.00	[0.00, 0.00]	Frontal_Mid_Orb2526
-0.06	[-0.14, 0.02]	Temporal_Sup
-0.03	[-0.07, 0.01]	Pallidum
-0.06	[-0.15, 0.02]	Insula
-0.06	[-0.15, 0.03]	Precentral
0.00	[-0.01, 0.00]	Fusiform
-0.06	[-0.16, 0.04]	Rolandic_Oper
0.00	[-0.01, 0.00]	ParaHippocampal
0.00	[-0.01, 0.00]	Supp_Motor_Area
0.00	[0.00, 0.00]	Occipital_Sup
-0.02	[-0.06, 0.02]	Hippocampus
0.00	[0.00, 0.00]	Occipital_Inf
0.00	[0.00, 0.00]	Cuneus
0.00	[0.00, 0.00]	Frontal_Sup_Medial
0.00	[0.00, 0.00]	Olfactory
0.00	[-0.01, 0.00]	Frontal_Inf_Tri
-0.01	[-0.02, 0.01]	Angular
-0.06	[-0.20, 0.07]	Thalamus
-0.01	[-0.02, 0.01]	Frontal_Sup
0.00	[0.00, 0.00]	Occipital_Mid
0.00	[0.00, 0.00]	Temporal_Inf
-0.01	[-0.05, 0.02]	Frontal_Inf_Oper
-0.01	[-0.04, 0.02]	Frontal_Mid
-0.01	[-0.02, 0.01]	Amygdala
0.00	[0.00, 0.00]	Lingual
0.00	[0.00, 0.00]	Calcarine
0.00	[0.00, 0.00]	Temporal_Pole_Mid
0.00	[-0.03, 0.03]	Temporal_Pole_Sup

Association Outcome ~Δ_{ChaCo}

Region	OR	95% CI
SupraMarginal	0.39	[0.20, 0.69]
Cingulum_Mid	0.20	[0.05, 0.63]
Postcentral	0.61	[0.37, 0.93]
Angular	0.63	[0.36, 0.95]
Insula	0.64	[0.39, 0.97]
Parietal_Inf	0.62	[0.36, 0.97]
Rolandic_Oper	0.67	[0.43, 0.99]
Precentral	0.68	[0.44, 0.99]
Temporal_Sup	0.61	[0.33, 0.97]
Frontal_Mid	0.60	[0.32, 0.96]
Frontal_Sup	0.54	[0.22, 0.95]
Frontal_Inf_Orb	0.65	[0.37, 1.00]
Frontal_Mid_Orb910	0.60	[0.31, 1.02]
Thalamus	0.75	[0.53, 1.04]
Fusiform	0.72	[0.43, 1.01]
Cingulum_Ant	0.38	[0.09, 1.01]
Frontal_Sup_Medial	0.00	[0.00, 0.57]
Caudate	0.78	[0.53, 1.10]
Hippocampus	0.77	[0.48, 1.07]
Temporal_Mid	0.76	[0.48, 1.10]
Calcarine	0.64	[0.30, 1.05]
Lingual	0.71	[0.32, 1.06]
Supp_Motor_Area	0.68	[0.34, 1.19]
ParaHippocampal	0.69	[0.31, 1.03]
Precuneus	0.78	[0.44, 1.10]
Frontal_Mid_Orb2526	0.42	[0.03, 1.17]
Cuneus	0.84	[0.55, 1.14]
Occipital_Mid	0.81	[0.46, 1.14]
Occipital_Inf	0.77	[0.34, 1.11]
Cingulum_Post	0.63	[0.21, 1.13]
Frontal_Inf_Tri	0.85	[0.56, 1.17]
Temporal_Inf	0.86	[0.57, 1.17]
Frontal_Sup_Orb	0.73	[0.33, 1.21]
Putamen	0.89	[0.64, 1.20]
Heschl	0.87	[0.58, 1.24]
Occipital_Sup	0.88	[0.56, 1.18]
Olfactory	0.86	[0.51, 1.28]
Frontal_Inf_Oper	0.92	[0.66, 1.25]
Pallidum	0.93	[0.69, 1.26]
Rectus	0.90	[0.45, 1.29]
Parietal_Sup	0.94	[0.66, 1.25]
Amygdala	0.98	[0.70, 1.35]
Paracentral_Lobule	1.01	[0.68, 1.40]
Temporal_Pole_Sup	1.04	[0.75, 1.43]
Temporal_Pole_Mid	1.23	[0.83, 1.77]

Supplementary Tab. 6: Selection of AAL regions for mediation analysis. Associations of change in connectivity with treatment (left) and favorable outcome (right) were assessed using linear and logistic regressions. Age, baseline NIHSS score and baseline lesion volume were included as covariates. Effect sizes were quantified by regression coefficients (beta) and odds ratios (OR). Regions were ranked by the statistical evidence for a non-vanishing association and considered a potential mediator if neither of the two 95% confidence intervals was compatible with the absence of an association. Matching regions are connected to indicate similarity of the two rankings. Color indicates anatomical assignment to frontal (cerulean), parietal (sun), temporal (dark cyan), occipital (summer sky) and limbic (violet red) lobes, or subcortical structures (orange).

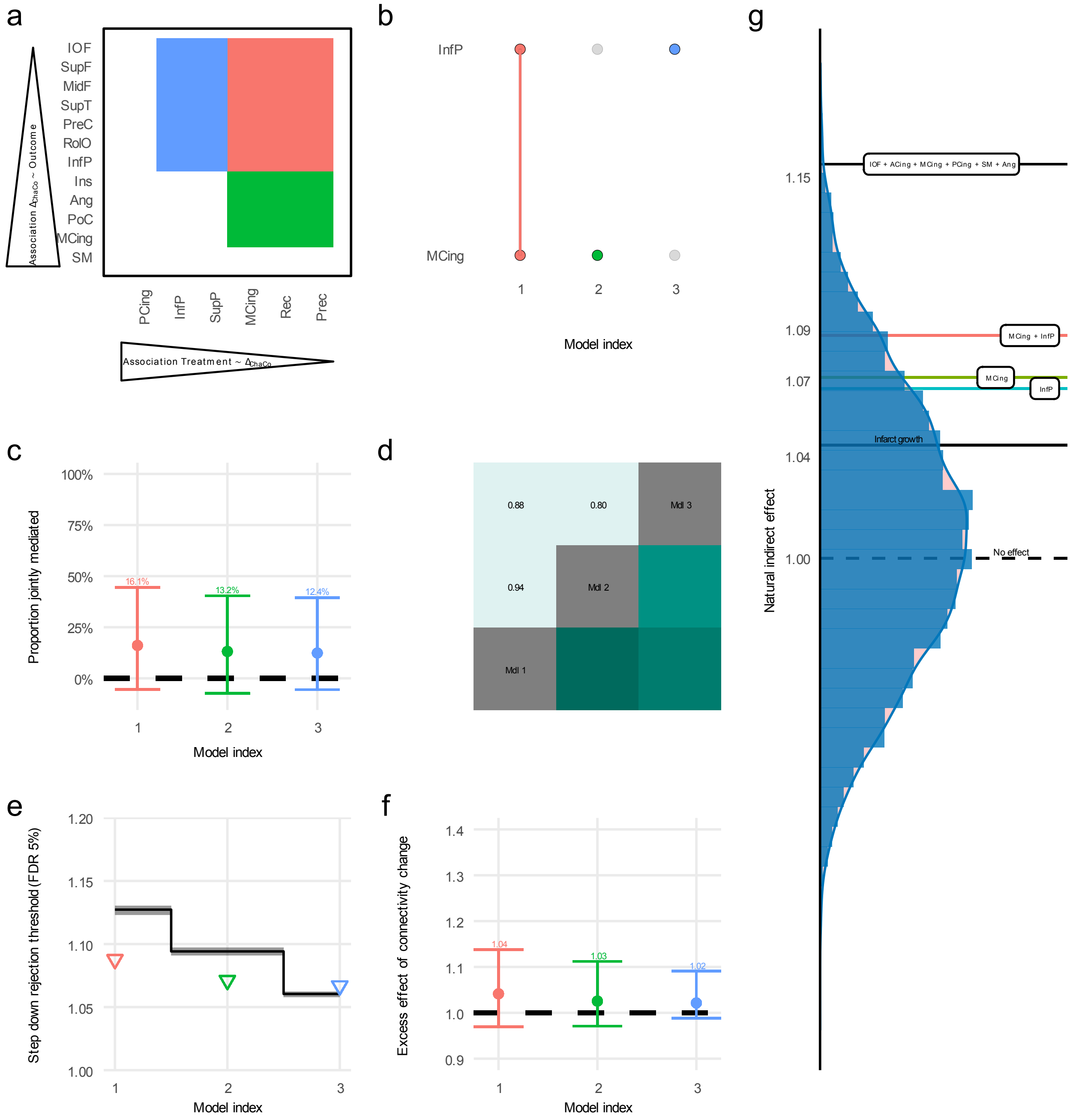

Supplementary Fig. 9. Results of AAL-based multiple mediation analysis, adjusted for age, baseline NIHSS score and pre-randomisation lesion volume. **a** Selection of brain regions by decreasing strength of association between treatment allocation and connectivity change (horizontal axis) and connectivity change and functional outcome (vertical axis). Each coloured patch corresponds to a mediation model including, as mediators, infarct growth as well as connectivity change in the intersection of the *i* most alteplase-responsive (horizontal axis) and *j* most clinically eloquent (vertical axis) regions. Identical colour coding is applied for the following subgraphs (b, c, e—g) to identify underlying referred models **b** Composition of resulting three mediation models comprising connectivity change in up to four brain regions. **c** Proportion of total effect of thrombolysis on favourable clinical outcome (mRS 0-1) mediated jointly through infarct growth and connectivity change in different sets of brain regions (n=263 independent subjects). Solid dots represent point estimates. Vertical bars indicate 95% bias-corrected confidence intervals obtained from bootstrap with k=10,000 replicates. **d** Empirical correlation between natural indirect effect estimates corresponding to different mediation models. Darker shades of green correspond to stronger correlations as indicated numerically in the left upper triangle of the matrix. **e** Step-down procedure to control the false discovery rate in the joint assessment of multiple mediation models at 5%. Models are ordered by their empirical natural indirect effect, indicated by triangles; beginning with the strongest effect, null hypotheses of no mediation effect are sequentially rejected as long as NIEs exceed their respective rejection threshold indicated by the black step line (median of k=100 bootstrap replicates). Shading indicates 95% confidence intervals for the rejection line. **f** Point estimates (solid dots) and 95% confidence intervals (vertical bars) for the ratio between joint natural indirect effects of infarct growth and connectivity loss and the NIE of infarct growth alone (bootstrap with k=10,000 replicates; n=263 independent subjects). **g** Null distribution of empirical natural indirect effects mediated jointly through infarct growth and connectivity change in random subsets of all brain regions. Horizontal lines indicated the mediation effects of infarct growth alone (black, NIE 1.04) and infarct growth in combination with connectivity change in alteplase-responsive clinically

eloquent areas (coloured). Manually defined model corresponding to the best-performing model from the DK-based analysis is shown in black.

NIE = Natural indirect effect, SM = supramarginal, MCing = mid cingulate, PoC = postcentral, Ang = angular gyrus, Ins = insula, InfP = Inferior parietal, RolO = Rolandic operculum, PreC = precentral, SupT = superior temporal, MidF = middle frontal, SupF = superior frontal, IOF = inferior orbital frontal, PCing = posterior cingulate, SupP = superior parietal, Rec = rectus, Prec = precuneus, ACing = anterior cingulate

Supplementary References

1. Brooks, M. E. *et al.* glmmTMB balances speed and flexibility among packages for zero-inflated generalized linear mixed modeling. *R J.* **9**, 378–400 (2017).

2. Healy, K. Book Review: An R and S-PLUS Companion to Applied Regression. *Sociol. Methods Res.* **34**, 137–140 (2005).

3. Kuceyeski, A., Maruta, J., Relkin, N. & Raj, A. The Network Modification (NeMo) Tool: Elucidating the Effect of White Matter Integrity Changes on Cortical and Subcortical Structural Connectivity. *Brain Connect.* **3**, 451–463 (2013).

4. Greenland, S. Valid P-Values Behave Exactly as They Should: Some Misleading Criticisms of P-Values and Their Resolution With S-Values. *Am. Stat.* **73**, 106–114 (2019).

5. Shannon, C. E. A Mathematical Theory of Communication. *Bell Syst. Tech. J.* **27**, 379–423, 623–656 (1948).

6. Holmes, C. J. *et al.* Enhancement of MR images using registration for signal averaging. *J. Comput. Assist. Tomogr.* **22**, 324–333 (1998).

7. Fischl, B. FreeSurfer. *Neuroimage* **62**, 774–781 (2012).
