# Supplementary material for: Preserved structural connectivity mediates the clinical effect of thrombolysis in patients with anterior-circulation stroke
Source: Nat Commun. 2021 May 10;12:2590. doi: 10.1038/s41467-021-22786-w (PMC8110812; doi:10.1038/s41467-021-22786-w)
Supplement: Supplementary file 2 — Reporting Summary [file 41467_2021_22786_MOESM2_ESM.pdf]

nature research

Corresponding author(s): Eckhard Schlemm

Last updated by author(s): Mar 4, 2021

Reporting Summary

Nature Research wishes to improve the reproducibility of the work that we publish. This form provides structure for consistency and transparency in reporting. For further information on Nature Research policies, see our Editorial Policies and the Editorial Policy Checklist.

Statistics

For all statistical analyses, confirm that the following items are present in the figure legend, table legend, main text, or Methods section.

n/a	Confirmed	
☐	☒	The exact sample size (*n*) for each experimental group/condition, given as a discrete number and unit of measurement
☐	☒	A statement on whether measurements were taken from distinct samples or whether the same sample was measured repeatedly
☐	☒	The statistical test(s) used AND whether they are one- or two-sided<br>*Only common tests should be described solely by name; describe more complex techniques in the Methods section.*
☐	☒	A description of all covariates tested
☐	☒	A description of any assumptions or corrections, such as tests of normality and adjustment for multiple comparisons
☐	☒	A full description of the statistical parameters including central tendency (e.g. means) or other basic estimates (e.g. regression coefficient) AND variation (e.g. standard deviation) or associated estimates of uncertainty (e.g. confidence intervals)
☐	☒	For null hypothesis testing, the test statistic (e.g. *F*, *t*, *r*) with confidence intervals, effect sizes, degrees of freedom and *P* value noted<br>*Give P values as exact values whenever suitable.*
☒	☐	For Bayesian analysis, information on the choice of priors and Markov chain Monte Carlo settings
☐	☒	For hierarchical and complex designs, identification of the appropriate level for tests and full reporting of outcomes
☐	☒	Estimates of effect sizes (e.g. Cohen's *d*, Pearson's *r*), indicating how they were calculated

Our web collection on statistics for biologists contains articles on many of the points above.

Software and code

Policy information about availability of computer code

Data collection	ClinCase (Quadratek Data Solutions Ltd) for clinical data, PACS for imaging data
Data analysis	SONIA v1.0<br>MATLAB v2019b, NeMo Toolbox v1 (https://github.com/jimmyshen007/NeMo)<br>R v4.0.2, with packages lme4_1.1-23, broom.mixed_0.2.6, glmmTMB_1.0.2.1, medflex_0.6-6

For manuscripts utilizing custom algorithms or software that are central to the research but not yet described in published literature, software must be made available to editors and reviewers. We strongly encourage code deposition in a community repository (e.g. GitHub). See the Nature Research guidelines for submitting code & software for further information.

Data

Policy information about availability of data

All manuscripts must include a data availability statement. This statement should provide the following information, where applicable:

- Accession codes, unique identifiers, or web links for publicly available datasets
- A list of figures that have associated raw data
- A description of any restrictions on data availability

All patient-level data used in the analysis, including age, baseline NIHSS score, lesion volumes, treatment allocation, connectivity measurements and functional outcome, are available on GitHub (https://github.com/csi-hamburg/WAKE-UP-preserved-SC). Imaging data, after de-identification, will be shared with the Virtual International Stroke Trials Archive (VISTA) and be accessible according to the VISTA rules (http://www.virtualtrialsarchives.org/vista).

Field-specific reporting

Please select the one below that is the best fit for your research. If you are not sure, read the appropriate sections before making your selection.

☒ Life sciences ☐ Behavioural & social sciences ☐ Ecological, evolutionary & environmental sciences

For a reference copy of the document with all sections, see nature.com/documents/nr-reporting-summary-flat.pdf

Life sciences study design

All studies must disclose on these points even when the disclosure is negative.

Sample size	503 patients randomized in WAKE-UP. Original planned sample size 880 patients based on expected 10% absolute difference in the proportion of favourable clinical outcome. Premature stop of enrollment due to cessation of funding.
Data exclusions	Exclusion of non-anterior-circulation and bilateral strokes. Exclusion of patients with missing follow-up imaging or imaging of insufficient quality for lesion segmentation. Final sample size n=269.
Replication	Analyses were performed with two different atlases (Desikan-Killiany and AAL). Replication in an independent sample of patients was not possible because of lack of data.
Randomization	Patients were randomized 1:1 to placebo or alteplase in four cohorts stratified according to age (<60, >60 years) and severity of symptoms (NIHSS <11, >10).
Blinding	Investigators were blinded during data collection and outcome assessment.

Reporting for specific materials, systems and methods

We require information from authors about some types of materials, experimental systems and methods used in many studies. Here, indicate whether each material, system or method listed is relevant to your study. If you are not sure if a list item applies to your research, read the appropriate section before selecting a response.

Materials & experimental systems

n/a	Involved in the study	
☒	☐	Antibodies
☒	☐	Eukaryotic cell lines
☒	☐	Palaeontology and archaeology
☒	☐	Animals and other organisms
☐	☒	Human research participants
☐	☒	Clinical data
☒	☐	Dual use research of concern

Methods

n/a	Involved in the study	
☒	☐	ChIP-seq
☒	☐	Flow cytometry
☐	☒	MRI-based neuroimaging

Human research participants

Policy information about studies involving human research participants

Population characteristics	Patients with acute ischemic stroke of unknown symptom onset in the anterior circulation with a mismatch between FLAIR und DWI on acute MR imaging.<br>Randomisation 1:1 to placebo or alteplase (intravenous thrombolysis)<br>Age: Placebo mean 66.0 ± 11.1 years; altepase mean 65.2 ± 11.3<br>Baseline NIHSS: Placebo median 7, interquartile range [4, 11]; alteplase median 6, IQR [3.5, 8]<br>Baseline lesion volume: Placebo 4.0 ml, IQR [1.3, 10] ml; alteplase 2.2 ml, IQR [0.87, 9.9] ml
Recruitment	Acute stroke patients were recruited by participating centers after confirmation of elegibility criteria. Patients for whom thrombectomy was planned, who had a very severy stroke (NIHSS score > 25) or large infarct (> 1/3 of the MCA territory), or had contraindications to treatment with altepase (other than unknown time of symptom onset) were excluded.
Ethics oversight	Ethics Advisory Board<br>Approval by Ethics Committees of all participating centres and national Regulatory Authorities (Belgium, Denmark, France, Germany, Spain, United Kingdom).

Note that full information on the approval of the study protocol must also be provided in the manuscript.

Clinical data

Policy information about clinical studies

All manuscripts should comply with the ICMJEguidelines for publication of clinical research and a completedCONSORT checklist must be included with all submissions.

Clinical trial registration	Post-hoc analysis of data from the WAKE-UP trial (ClinicalTrials.gov number NCT01525290, EudraCT number 2011-005906-32). ICMJE guideline and CONSORT checklist not applicable.
Study protocol	WAKE-UP: https://www.nejm.org/doi/suppl/10.1056/NEJMoa1804355/suppl_file/nejmoa1804355_appendix.pdf
Data collection	Multicentric patient recruitment across 70 European sites Sep 2012 -- Jun 2017. Analysis Nov 2018 -- May 2020
Outcomes	Primary outcome modified Rankin scale score 0-1 90 days after randomisation, clinical assessment

Magnetic resonance imaging

Experimental design

Design type	Structural imaging at rest. Clinical MRI including FLAIR and DWI
Design specifications	Two imaging sessions per patient: before randomisation and 22-36 hours post-randomisation
Behavioral performance measures	No behavioural performance measures recorded during the scan. Functional outcome after 90 days measured on the modified Ranking scale.

Acquisition

Imaging type(s)	structural
Field strength	1.5 T
Sequence & imaging parameters	DWI: Spin-echo EPI, TR >= 4000ms, b=0 and 1000 sec/mm^2, imge duration 90-260 sec, >=12 slices, slice thickness 5 mm, gap 0-1 mm, matrix size 128x128, phase-encoding along A/P direction, field of view 24 cm (https://doi.org/10.1161/STROKEAHA.107.512319)
Area of acquisition	whole brain

Diffusion MRI ☐ Used ☒ Not used

Preprocessing

Preprocessing software	SONIA v1.0 (in house)
Normalization	non-linear, FLAIR
Normalization template	MNI152
Noise and artifact removal	n/a (single volume per subject, session and sequence)
Volume censoring	n/a (single volume per subject, session and sequence)

Statistical modeling & inference

Model type and settings	first-level modelling: not applicable to FLAIR / DWI data<br>second-level modelling: mixed-effects generalised linear regressions, natural effects mediation using inverse weighting
Effect(s) tested	Association loss of connectivity ~ treatment allocation (alteplase, placebo)<br>Association functional outcome (mRS <= 1) ~ loss of connectivity<br>Causal mediation

Specify type of analysis: ☐ Whole brain ☒ ROI-based ☐ Both

Anatomical location(s): Desikan-Killiany atlas

Statistic type for inference (See Eklund et al. 2016)	n/a. No voxelwise or clusterwise analysis performed.
Correction	For the association loss of connectivity ~ treatment allocation (alteplase, placebo) a single main hypothesis is tested (Tab. 1). For mediation analysis, several models are considered; here correction for multiple comparison is achieved using bootstrap to control the FDR.

Models & analysis

n/a	Involved in the study
☒	☐ Functional and/or effective connectivity
☒	☐ Graph analysis
☒	☐ Multivariate modeling or predictive analysis
